# Selective Anti-Giardial Action of Indirubin: Biochemical and Functional Evidence for Inhibition of Triosephosphate Isomerase and Aldose Reductase in *Giardia lamblia*

**DOI:** 10.3390/ijms27104167

**Published:** 2026-05-07

**Authors:** Luis Antonio Flores-López, Gabriela López-Herrera, Yoalli Martínez-Pérez, Elías Jaime Matadamas-Ortiz, Saúl Gómez-Manzo, Gloria Hernández-Alcántara, Angélica González-Maciel, Rafael Reynoso-Robles, Beatriz Hernández-Ochoa, Laura Chino-Ríos, Diego González-Gómez, Leonardo Valente Arteaga-Padilla, Sergio Enríquez-Flores, Ignacio De la Mora-de la Mora

**Affiliations:** 1 Laboratorio de Biomoléculas y Salud Infantil, SECIHTI—Instituto Nacional de Pediatría, Mexico City 04530, Mexico; luisbiolexp@gmail.com; 2 Laboratorio de Inmunodeficiencias Primarias, Instituto Nacional de Pediatría, Secretaría de Salud, Mexico City 04530, Mexico; lohegabyqbp@gmail.com; 3 Escuela de Medicina y Ciencias de la Salud, Tecnológico de Monterrey, Mexico City 14380, Mexico; yoalli.martinez@tec.mx; 4 Laboratorio de Investigación de Agro-Recursos y Metabolitos Secundarios, Universidad Autónoma Chapingo, Texcoco 56230, Mexico; emata993@hotmail.com; 5 Laboratorio de Bioquímica Genética, Instituto Nacional de Pediatría, Secretaría de Salud, Mexico City 04530, Mexico; saulmanzo@ciencias.unam.mx; 6 Departamento de Bioquímica, Facultad de Medicina, Universidad Nacional Autónoma de México, Mexico City 04510, Mexico; ghernandez@bq.unam.mx; 7 Laboratorio Morfología Celular y Tisular, Instituto Nacional de Pediatría, Secretaría de Salud, Mexico City 04530, Mexico; agonzalezmaciel@yahoo.com (A.G.-M.); reynosoraf@yahoo.com (R.R.-R.); 8 Laboratorio de Investigación en Ciencias Ómicas y Epidemiología Microbiana, Hospital Infantil de México Federico Gómez, Secretaría de Salud, Mexico City 06720, Mexico; beatrizhb_16@comunidad.unam.mx; 9 Posgrado en Ciencias Biológicas (Maestría), Universidad Nacional Autónoma de México, Mexico City 04510, Mexico; laura-cr@ciencias.unam.mx; 10 Laboratorio de Biomoléculas y Salud Infantil, Instituto Nacional de Pediatría, Secretaría de Salud, Mexico City 04530, Mexico; 11 Licenciatura en Bioquímica Diagnóstica, Facultad de Estudios Superiores Cuautitlán, Universidad Nacional Autónoma de México, Mexico City 04510, Mexico; 318094414@cuautitlan.unam.mx (D.G.-G.); 318173580@cuautitlan.unam.mx (L.V.A.-P.)

**Keywords:** indirubin, dual effect, metabolic inhibition, dimethylsulfoxide, carbonyl stress, methylglyoxal

## Abstract

The emergence of clinical resistance to conventional antigiardials underscores the need for compounds with novel mechanisms of action. This study demonstrates that indirubin exerts antigiardial activity by targeting important metabolic enzymes in *Giardia lamblia*. Indirubin induced a concentration-dependent decrease in trophozoite proliferation and viability, correlating with reduced activity of native triosephosphate isomerase and aldose reductase. Using recombinant enzymes, indirubin directly inhibited triosephosphate isomerase and aldose reductase, with the parasite enzymes showing greater susceptibility than their human orthologs. Structural and computational analyses suggest preferential binding of indirubin at the dimer interface of triosephosphate isomerase and within the NADP(H)-binding pocket of aldose reductase. The dual enzymatic inhibition was concordant with methylglyoxal accumulation, extensive protein carbonylation, and the formation of advanced glycation end products. These effects culminated in apoptotic-like death and severe ultrastructural damage, including alteration of the adhesive disc and microtubule networks. By targeting vulnerabilities in the metabolic and redox pathways of *G. lamblia* through a multifactorial mechanism distinct from current therapies, our findings support indirubin as a promising candidate for the treatment of giardiasis.

## 1. Introduction

*Giardia lamblia* (also known as *Giardia duodenalis*) is a microaerophilic protozoan parasite of the human gastrointestinal tract and a major cause of chronic diarrheal and post-infectious gastrointestinal disease worldwide [1]. This microorganism represents an increasing threat to public health, particularly in developing countries, with infections affecting more than 200 million people annually [1,2].

The management of human giardiasis currently relies on a small number of approved drug classes [3]. For the past four decades, first-line therapy has consisted of nitroheterocyclics, such as metronidazole and tinidazole [3]. However, these drugs are often associated with several side effects [1]. Furthermore, a significant proportion of clinical cases now involve metronidazole- and tinidazole-resistant *G. lamblia* [4], and the recently reported increase in resistance to these drugs worldwide is a cause for concern [5].

Second-line drugs, including albendazole, nitazoxanide, furazolidone, and paromomycin, are available but generally have lower efficacy and potential side effects [4,6]. These limitations, combined with the adverse effects associated with existing treatments, underscore the inadequacy of current therapeutic options and guide the search for new pharmacological targets [1,2].

Understanding the mechanisms of drug action and resistance is critical to developing new therapies. Nitroheterocyclics are redox-active compounds that are thought to damage proteins and DNA after activation by oxidoreductase enzymes in metabolically active cells, although the exact mechanisms of cell death remain unknown. As providers of reducing power, glycolytic enzymes and oxidoreductases actively participate in both the activation and detoxification of these drugs, a dual role that contributes to the emergence of resistant strains. Nitroheterocyclic-resistant *G. lamblia* has been isolated from patients and developed in vitro, prompting considerable research into the biomolecular mechanisms of resistance [3].

The growing need for new and effective drugs with novel mechanisms of action arises from this increasing drug resistance. In this context, parasite-specific metabolic vulnerabilities represent attractive alternatives. *G. lamblia* relies heavily on glycolysis for ATP production and on oxidoreductase systems for redox balance and detoxification. Therefore, enzymes involved in energy metabolism and oxidative stress management constitute rational drug targets.

Triosephosphate isomerase from *G. lamblia* (GlTPI) is a central glycolytic enzyme required for efficient ATP generation. Previous studies have demonstrated that GlTPI can be selectively targeted and its inhibition correlates with toxic effects on the parasite [7,8]. Structurally, TPI functions as a homodimer, and disruption of its dimer interface has emerged as a strategy to achieve selectivity over the human ortholog.

In the search for new drug targets, attention has also turned to enzymes essential for the parasite’s unique energy metabolism. *G. lamblia* aldose reductase (GlAR) is an important enzyme in this pathway, specifically in the production of ethanol, and plays a vital role in parasite survival and growth. It contributes to both energy production and detoxification by participating in NADP(H)-dependent redox reactions linked to cellular homeostasis. Despite its importance, little is known about this enzyme. However, sodium valproate has been shown to inhibit GlAR, thereby reducing ethanol production and trophozoite growth [9]. Furthermore, an in silico study of the diterpene compound Linearolactone (LL) reported significant giardicidal activity, demonstrating its ability to induce pro-necrotic death in the parasite. Molecular docking analysis suggested that LL interacts with GlAR, proposing this enzyme as a likely pharmacological target [10].

On the other hand, the search for effective enzyme inhibitors has gained significant importance, with natural phytochemicals emerging as promising candidates. These compounds, derived from diverse plant sources, offer structural complexity, bioavailability, and the potential to interact selectively with Aldose reductase (AR) [11].

Indirubin (IND) and its derivatives have been shown to inhibit human glycogen synthase kinase 3β and CDK5/p25 [12] and have potential inhibitory action against cyclin-2 dependent kinase (CDK-2) [13]. A single report on *G. lamblia* identified IND as a trophozoite proliferation agonist through high-throughput screening, concluding that 35–75 μM promoted parasite growth [14].

Given the urgent priority to identify new targeted therapies that are safe, cost-effective, and orally bioavailable for the effective treatment of giardiasis [15], this study seeks to provide new insights into the inhibition of energetic and detoxification enzymes through the use of the natural compound IND, an organic drug with proven therapeutic potential in other pathologies.

Our data compares the effect of the vehicle dimethylsulfoxide (DMSO) and IND on cell proliferation and viability. This was done in conjunction with monitoring the residual activities of native and recombinant enzymes of the parasite *G. lamblia* vs. the treatments.

We used the following strategies to determine the lethal effect of IND on *G. lamblia*. Three concentrations of the compound were used to assess cell proliferation and death in *G. lamblia*. Subsequently, the residual activities of the parasite’s native enzymes, as well as the recombinant enzymes GlTPIr and GlARr, were determined against their human orthologs HsTPIr and HsAKR1-A1r. The formation of hydrophobic patches resulting from exposure to the compound was assessed by extrinsic fluorescence in both *G. lamblia* enzymes. Methylglyoxal (MGO) and advanced glycation end products (AGEs) levels were determined in the treated parasites. Flow cytometry of the parasites provided evidence of the IND-induced apoptotic-like cell death. The effect of IND on the ultrastructure of the parasites was corroborated by transmission electron microscopy (TEM), and finally the binding site of IND and its affinity towards both enzymes analyzed was determined by molecular docking to their crystallographic structures.

## 2. Results

### 2.1. Effects of IND on Giardia lamblia Trophozoite Proliferation and Viability

Given the urgent need to identify alternative antigiardial compounds with novel mechanisms of action and considering previous reports describing both proliferative and inhibitory effects of IND on *G. lamblia*, the effect of increasing IND concentrations on trophozoite proliferation and viability in vitro was evaluated. These experiments were designed to determine whether IND exerts cytostatic and/or cytotoxic effects on this organism. Importantly, all cellular and enzymatic responses were interpreted relative to their corresponding DMSO vehicle controls, as the solvent concentration was kept constant across all IND treatments. This normalization is relevant and supports that the differences observed between the conditions are attributable to IND and not to variations in exposure to solvents.

**Figure 1:**
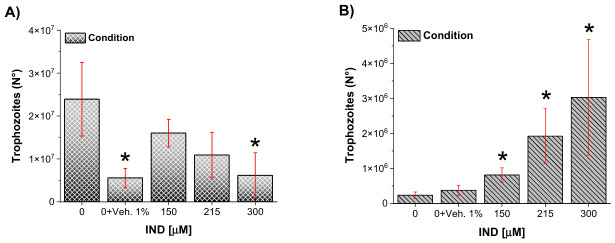
**Effect of IND on the proliferation and viability of *Giardia lamblia* trophozoites after 72 h of incubation.** Trophozoites were exposed to the compound at 0, 150, 215, and 300 µM, including a vehicle control corresponding to the highest DMSO concentration used (1%). (**A**) Total trophozoite counts per experimental condition. (**B**) Comparative analysis of trophozoite cell death determined by trypan blue exclusion under each condition. Data correspond to three independent biological replicates and are presented as the mean, with bars indicating experimental standard deviation. Statistical analysis was performed using one-way ANOVA followed by Tukey’s post hoc test, with statistical significance defined as *p* < 0.05 (* with respect to the corresponding control).

After 72 h of incubation, trophozoite proliferation was quantified by cell counting. Control cultures reached an average density of almost 24 × 10^6^ cells, whereas cultures exposed to the vehicle control (1% DMSO) showed a reduced cell number of 5.55 × 10^6^ cells (Figure 1A). On the other hand, exposure to IND resulted in a concentration-dependent decrease in trophozoite proliferation, with average cell counts of 16.01-, 10.88-, and 6.13 × 10^6^ cells at 150, 215, and 300 µM IND, respectively (Figure 1A).

To further assess whether the reduction in trophozoite number was associated with loss of viability, cell death was evaluated using the trypan blue exclusion assay. Basal cell death in control cultures was minimal (0.1%). In comparison, the vehicle control exhibited 6.83% nonviable trophozoites (Figure 1B). IND treatment induced a concentration-dependent increase in cell death, with 5.9%, 17.67%, and 49.44% dead cells observed at 150, 215, and 300 µM, respectively (Figure 1B). The pronounced increase in nonviable trophozoites at the highest IND concentration is consistent with the notable reduction in cell numbers observed under these conditions.

### 2.2. IND Reduces Trophozoite Proliferation and Viability in Parallel with Decreased Native GlTPI and GlAR Activities

Because glycolytic and detoxification enzymes play central roles in *G. lamblia* energy metabolism and redox homeostasis and have been implicated in activation and resistance mechanisms to antigiardial drugs, it was evaluated whether IND affects the activity of TPI and AR enzymes in trophozoite extracts. These experiments were designed to assess whether decrease in trophozoite proliferation and viability correlates with alterations in the activity of enzymes involved in energetic and detoxification pathways.

**Figure 2:**
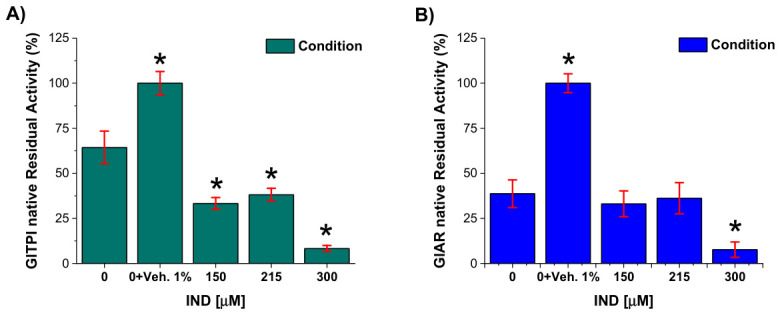
**Residual activity of the cellular enzymes TPI and AR in *Giardia lamblia* trophozoites following treatment with increasing concentrations of IND.** Enzymatic activities were measured in cell lysates as described in the Methods section and are expressed relative to the corresponding vehicle control. (**A**) TPI residual activity. (**B**) AR residual activity. Data correspond to three independent biological replicates and are presented as the mean, with bars indicating experimental standard deviation. Statistical analysis was performed using one-way ANOVA followed by Tukey’s post hoc test, with statistical significance defined as *p* < 0.05 (* with respect to the corresponding control).

To address this, trophozoites were exposed to increasing concentrations of IND for 72 h, after which cells were lysed and the activities of the native enzymes TPI and AR were quantified as described in the Methods section. In both cases, the DMSO control showed higher enzymatic activity compared with the DMSO-free control. Notably, in the absence of IND, TPI activity in the control condition was approximately 40% lower than in the vehicle control, whereas AR activity showed a more pronounced reduction of approximately 60% relative to the vehicle (Figure 2A,B; Supplementary Table S1).

Exposure to IND was associated with a concentration-dependent decrease in the activity of both enzymes. At 215 and 300 µM IND, TPI activity was reduced by approximately 60–90% relative to the vehicle control (Figure 2A). A similar trend was observed for AR, with activity reductions of approximately 60% and 90% at 215 and 300 µM IND, respectively (Figure 2B). These reductions in enzymatic activity were observed under experimental conditions in which decreased trophozoite proliferation and increased cell death were also detected.

### 2.3. Effect of Vehicle (DMSO) Exclusion on Native TPI and AR Activities

Given that *G. lamblia* metabolism is highly susceptible to oxidative stress and that both TPI and AR contain cysteine residues critical to their catalytic functions, it is imperative to distinguish the inhibitor’s pharmacological effect from potential experimental artifacts. Since DMSO, used as a vehicle for IND, has been implicated in the oxidation of thiol groups [16], its presence poses a risk of confounding the interpretation of residual enzymatic activity. Therefore, to ensure the internal validity of the assays and confirm direct inhibition by IND, a background-subtraction correction was performed to isolate the compound’s net effect on the native enzymes.

**Figure 3:**
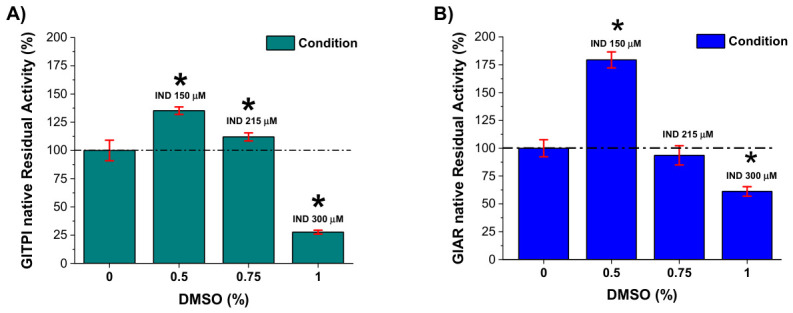
**Residual activity of intracellular enzymes after correction for DMSO effects.** TPI (**A**) and AR (**B**) activities were measured in *G. lamblia* trophozoite protein extracts following 72 h exposure to IND at 150, 215, and 300 μM at 37 °C. Residual activities obtained from vehicle-treated samples (0.5%, 0.75%, and 1% DMSO, respectively) were subtracted from IND-treated samples. Data represent the enzymatic activity attributable specifically to IND after vehicle exclusion. Data correspond to three independent biological replicates and are presented as the mean, with bars indicating experimental standard deviation. Statistical analysis was performed using one-way ANOVA followed by Tukey’s post hoc test, with statistical significance defined as *p* < 0.05 (* with respect to the corresponding control).

Therefore, trophozoite protein extracts were exposed to DMSO at concentrations equivalent to those used to dissolve IND (0.5%, 0.75%, and 1%), corresponding to IND concentrations of 150, 215, and 300 μM, respectively. The residual enzymatic activity measured in these vehicle-treated samples was considered background. These values were subtracted from the residual activity obtained in the corresponding IND-treated native protein samples. After background subtraction, the net activities of native TPI and AR are shown in Figure 3A,B. Both enzymes exhibited a concentration-dependent decrease in residual activity upon IND treatment. Importantly, this correction confirms that the observed reduction in enzymatic activity is attributable to IND and not to DMSO-induced oxidative effects, thereby supporting a direct inhibitory effect of the compound on intracellular TPI and AR.

### 2.4. Direct, Time-Dependent, and Solvent-Validated Effects of IND on Recombinant GlTPI, GlAR, and Their Human Counterparts

To determine whether the enzymatic alterations observed in trophozoite extracts were the consequence of a direct enzyme–compound interaction, purified recombinant GlTPIr and GlARr were incubated with increasing concentrations of IND under controlled in vitro conditions. Recombinant human TPI (HsTPIr) and human aldo-keto reductase-1 A1 (HsAKR1-A1r) were analyzed in parallel to evaluate species selectivity. Residual enzymatic activity was quantified following 2 h and 24 h of incubation to distinguish early versus sustained effects.

The use of purified recombinant proteins demonstrated that IND exerts its effects independently of additional cellular components, confirming a direct enzyme–compound interaction. All activity changes described below therefore reflect each enzyme’s intrinsic susceptibility to IND.

**Figure 4:**
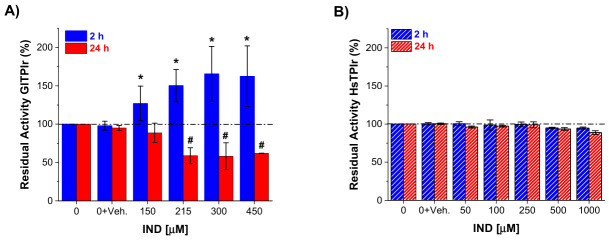

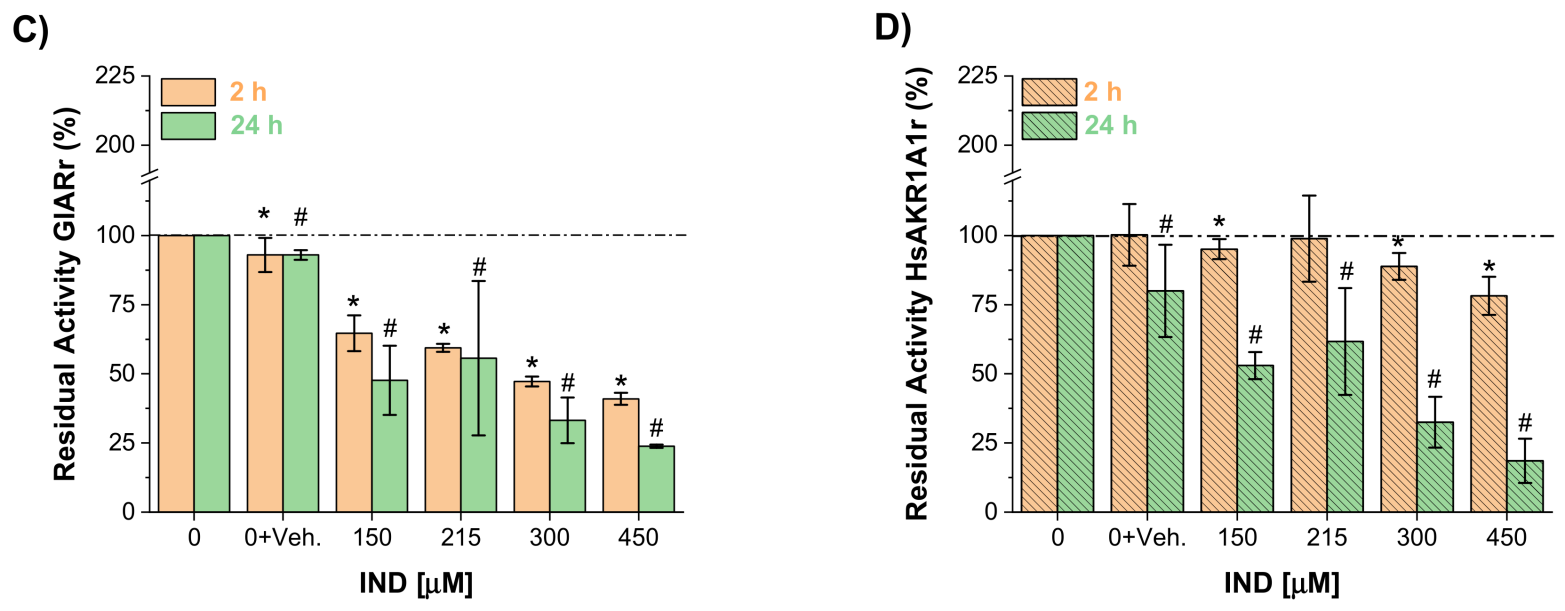
**Time- and concentration-dependent effects of IND on the residual catalytic activity of recombinant *Giardia lamblia* and human enzymes.** (**A**) GlTPIr and (**B**) HsTPIr were incubated at 0.2 mg/mL with IND (0–1000 μM) in 100 mM triethanolamine buffer, pH 7.4. (**C**) GlARr and (**D**) HsAKR1-A1r were incubated at 0.5 mg/mL with IND (0–450 μM) in 100 mM NaH_2_PO_4_ buffer, pH 7.0. Residual enzymatic activity was determined after 2 h (early exposure) and 24 h (prolonged exposure) of incubation. Data correspond to three independent biological replicates and are presented as the mean, with bars indicating experimental standard deviation. Statistical analysis was performed using one-way ANOVA followed by Tukey’s post hoc test, with statistical significance defined as *p* < 0.05 (*, # with respect to the corresponding control).

After 2 h of incubation, GlTPIr exhibited a reproducible increase in apparent residual activity beginning at 150 μM IND and reaching approximately 127–166% relative to untreated controls (Figure 4A, blue bars). This transient stimulation is consistent with previous report describing enhanced trophozoite proliferation at low IND concentrations [14], and correlates with the elevated GlTPIr activity detected in trophozoites exposed to 150 μM IND for 72 h. These findings indicate that, at early time points, IND promotes a functional enhancement of the parasitic enzyme. However, prolonged exposure (24 h) reversed this effect. GlTPIr activity decreased in a concentration-dependent manner, with approximately 50% loss of residual activity between 215 and 450 μM IND relative to control (Figure 4A, red bars). Thus, the initial activation is transient and precedes progressive enzymatic inactivation. This biphasic behavior reveals a complex interaction in which IND initially enhances catalytic performance but ultimately impairs function upon sustained exposure.

To explore the mechanism underlying the early activation of GlTPIr, additional experiments were performed using intrinsic tryptophan fluorescence as a structural probe (Supplementary Figure S1). Recombinant GlTPI was incubated with IND (450 µM), and fluorescence spectra were recorded at 0, 30, 90, and 150 min. At time zero, IND induced a pronounced decrease in enzyme fluorescence intensity near to 60% of the control (buffer only), while DMSO alone produced a slight increase near to 110%. This quenching indicates a rapid conformational rearrangement altering the microenvironment of tryptophan residues. The fluorescence signal remained stable at 55–53% of control at 30, 90, and 150 min, strongly suggesting that the conformational change is established immediately and sustained over the early time window corresponding to enzymatic activation (127–166% at 2 h). These results support the interpretation that IND promotes a stable, catalytically favorable conformational state rather than nonspecific destabilization (Supplementary Figure S1).

Importantly, HsTPIr remained largely unaffected under equivalent conditions. From 2 h to 24 h of incubation, even at concentrations up to 1 mM IND, only minimal changes in residual activity were observed (Figure 4B, blue and red bars). This marked differential susceptibility between the parasitic and human enzymes highlights strong species selectivity at the TPI level.

In contrast to GlTPIr, GlARr did not display an early stimulation phase. Instead, IND induced direct inhibition at short incubation times. After 2 h, 450 μM IND reduced GlARr residual activity by approximately 60% relative to control (Figure 4C, cream bars). Following 24 h, inhibition was more pronounced, reaching approximately 76% loss of activity at the same concentration (Figure 4C, green bars), indicating progressive, time-dependent inactivation.

Evaluation of the human homolog HsAKR1-A1r revealed a distinct temporal profile. At 2 h, IND caused only a modest reduction in activity (~22% at 450 μM; Figure 4D, cream bars), suggesting lower early sensitivity compared with GlARr. However, after 24 h of exposure, HsAKR1-A1r exhibited substantial inhibition (~78% loss of activity at 450 μM; Figure 4D, green bars), approaching the degree of inactivation observed for the parasitic enzyme. This delayed susceptibility indicates reduced short-term sensitivity but raises the possibility of long-term off-target effects.

To validate that the observed enzymatic effects were not driven by solvent–compound interactions, complementary assays were performed using ethanol as an alternative vehicle (Supplementary Figure S2A). Ethanol alone exhibited intrinsic, concentration- and time-dependent effects on enzyme activity. For GlTPIr, ethanol produced progressive inhibition at concentrations of 1% and above (2 h: 70% and 60% residual activity at 1% and 1.5%, respectively; 24 h: 65% and 57%). Under these conditions, IND dissolved in 1.5% ethanol resulted in residual activities generally around one-half of control at both time points, without a clear dose–response relationship and closely resembling the inhibitory effect of ethanol alone.

Similarly, for GlARr, ethanol alone induced slight activation at short incubation times (2 h: 105–108% at 0.5–0.75%) but caused progressive inhibition at 24 h (down to about 70% at 1.5%). In the presence of IND (1.5% ethanol), residual activity remained high at 2 h (approximately 93–98%) and decreased at 24 h (about 66–92%), again without a consistent dose-dependent pattern attributable to IND. These findings indicate that ethanol significantly affects enzyme activity and obscures ligand-specific effects.

Importantly, under ethanol-based conditions, IND did not exhibit a clear or reproducible activity profile beyond the intrinsic solvent effect, likely due to its limited solubility and scarce effective bioavailability. In contrast, in DMSO-based assays, solvent controls showed negligible effects while IND produced well-defined, concentration-dependent responses. Full datasets are provided in (Supplementary Figure S2B).

### 2.5. IND Induces Time-Dependent Conformational Rearrangements Associated with Hydrophobic Surface Exposure

To determine whether structural alterations accompanied the functional inhibition observed for GlTPIr and GlARr, conformational changes were monitored using the hydrophobic probe ANSA. This fluorophore increases in intensity upon binding to hydrophobic regions that are normally buried within the protein core; thus, enhanced fluorescence reflects exposure of hydrophobic patches typically associated with unfolding or structural rearrangement.

**Figure 5:**
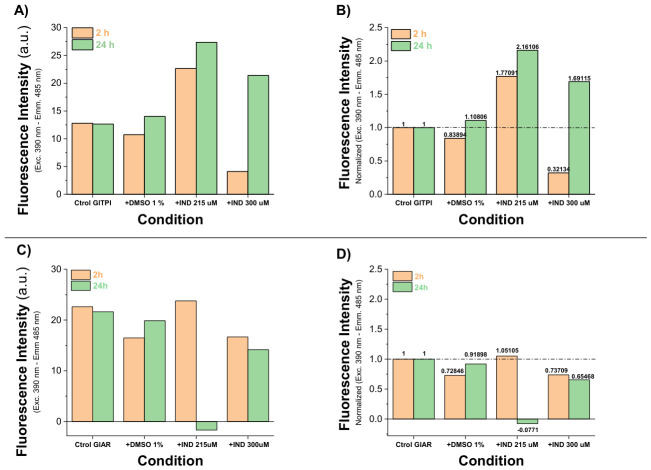
**IND-induced conformational changes assessed by ANSA extrinsic fluorescence in recombinant *Giardia lamblia* enzymes.** (**A**,**B**) GlTPIr and (**C**,**D**) GlARr were incubated under the same experimental conditions as in the enzymatic assays to directly correlate structural alterations with enzymatic activity. GlTPIr (0.2 mg/mL) and GlARr (0.5 mg/mL) were incubated in their respective buffers in the absence or presence of vehicle (DMSO) and IND for 2 h and 24 h. Following incubation, samples were exposed to ANSA (160 μM). Fluorescence emission was recorded at λex 390 nm and λem 485 nm using a spectrofluorometer. Panels A and C present fluorescence intensity in arbitrary units (a.u.), reflecting the extent of hydrophobic surface exposure. Panels B and D show normalized fluorescence values relative to the corresponding time-matched control (set to 1). Blank signals were subtracted from all measurements.

Because DMSO can interact with proteins via hydrogen bonding and, under certain conditions, promote oxidative modifications of sulfhydryl groups [17], its contribution to structural perturbation was first evaluated. In agreement with the enzymatic assays, 1% DMSO produced only marginal changes in ANSA fluorescence for both GlTPIr and GlARr (Figure 5A,C). These minimal effects confirm that the solvent does not significantly alter protein conformation under the experimental conditions, and that subsequent structural changes can be attributed to IND.

Thus, recombinant GlTPIr was incubated with 215 or 300 μM IND, and ANSA fluorescence was measured at 2 h and 24 h.

At 215 μM IND, ANSA fluorescence increased approximately 1.7-fold relative to control, indicating enhanced exposure of hydrophobic regions and suggesting partial unfolding or conformational “opening” of the enzyme. In contrast, at 300 μM IND, ANSA fluorescence decreased sharply to ~0.3-fold relative to control. This reduction is consistent with a distinct structural state, possibly reflecting rapid structural collapse or early aggregation, thereby restricting probe accessibility.

After 24 h, structural perturbation became more pronounced. At 215 μM IND, fluorescence increased further (from 1.77- to 2.16-fold vs. control), indicating progressive exposure of hydrophobic regions over time. Notably, at 300 μM IND, fluorescence rose dramatically compared with its 2 h value (from 0.32- to 1.69-fold vs. control), representing a 5.3-fold increase relative to the same condition at 2 h. This delayed increase suggests that initial structural compaction or aggregation is followed by progressive unfolding or destabilization.

Importantly, these time-dependent conformational changes parallel the loss of catalytic activity observed at 24 h (Figure 5A), supporting a direct structure–function correlation. The progressive exposure of hydrophobic regions indicates that IND acts as a structural destabilizer of GlTPIr, promoting conformations incompatible with catalytic competence. Thus, enzymatic inactivation appears to result from substantial structural disruption consistent with partial denaturation. DMSO alone produced only a slight (~10%) increase in ANSA fluorescence at 24 h, confirming minimal solvent contribution. However, at 2 h, 215 μM IND induced a 1.77-fold increase in fluorescence, which further rose to 2.16-fold at 24 h, indicating sustained exposure of hydrophobic regions. At 300 μM, fluorescence initially decreased (~0.3-fold at 2 h) but increased to ~1.69-fold relative to control after 24 h.

However, unlike GlTPIr, these fluorescence changes did not indicate extensive global unfolding. Instead, they coincided with progressive enzymatic inactivation (Figure 5C) without evidence of dramatic structural collapse. The modest yet consistent alterations in hydrophobic exposure suggest localized conformational perturbations, potentially near or within the catalytic site.

The time-dependent nature of inactivation, together with these structural observations, is compatible with a slow-binding mechanism in which IND progressively stabilizes an inactive enzyme conformation. Rather than inducing large-scale denaturation, IND appears to promote subtle structural rearrangements that impair catalytic function.

These data establish IND as a direct structural disruptor of essential parasitic enzymes, forcing them into conformations incompatible with sustained catalytic activity and, consequently, with parasite viability.

### 2.6. IND Triggers Carbonyl Stress and AGEs Accumulation in Giardia lamblia Trophozoites

Because GlTPI is a central glycolytic enzyme and GlAR participates in NADP(H)-dependent detoxification pathways, their inhibition is predicted to disrupt carbon flux and redox homeostasis in *G. lamblia*. Reduced TPI activity favors the non-enzymatic degradation of triosephosphate intermediates (G3P and DHAP) into MGO, a highly reactive dicarbonyl compound [18]. Simultaneous impairment of GlAR activity may further compromise NADP(H)-dependent detoxification capacity, thereby limiting the parasite’s ability to neutralize reactive carbonyl species.

Based on this framework, we hypothesized that IND-induced inhibition of GlTPI and GlAR would provoke intracellular carbonyl stress, leading to MGO accumulation and the secondary formation of AGEs. To test this, intracellular levels of MGO and AGEs were quantified in trophozoites exposed to IND for 72 h.

**Figure 6:**
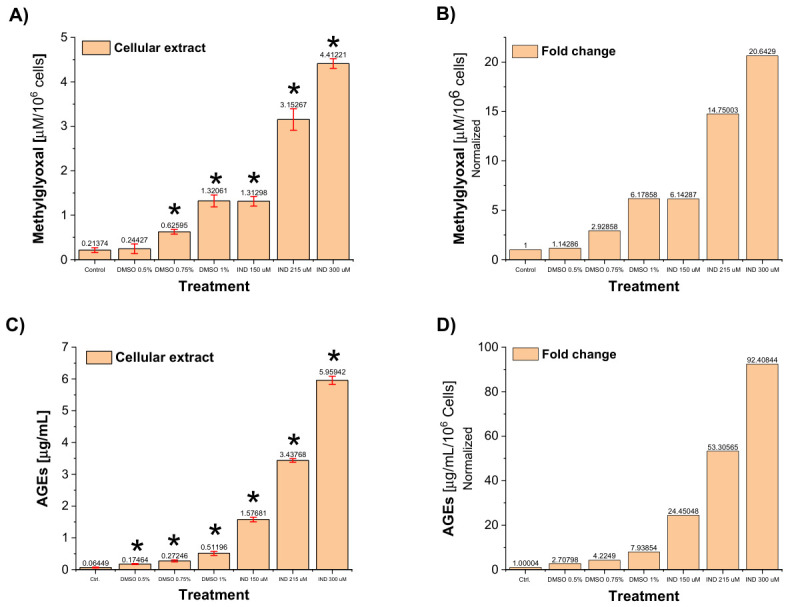
**IND promotes carbonyl stress and AGE accumulation in *Giardia lamblia* trophozoites.** Trophozoites were treated for 72 h at 37 °C with increasing concentrations of IND (150, 215, and 300 μM), including the corresponding DMSO vehicle controls at each concentration. After treatment, cells were lysed and processed for quantification of MGO and AGEs. (**A**) Intracellular MGO levels expressed as μM per 10^6^ trophozoites. (**B**) MGO levels normalized to the untreated control (set to 1). IND induced a concentration-dependent increase in MGO, reaching ~4.4 μM per 10^6^ cells at 300 μM IND, corresponding to an approximate 20.6-fold increase relative to control. (**C**) AGEs levels expressed as μg/mL. (**D**) AGEs levels normalized to the untreated control (set to 1). Data correspond to three independent biological replicates and are presented as the mean, with bars indicating experimental standard deviation. Statistical analysis was performed using one-way ANOVA followed by Tukey’s post hoc test, with statistical significance defined as *p* < 0.05 (* with respect to the corresponding control).

Protein extracts from trophozoites treated with 150, 215, and 300 μM IND displayed a clear concentration-dependent increase in MGO levels. Absolute concentrations reached 1.3, 3.15, and 4.41 μM per 10^6^ cells, respectively (Figure 6A). After subtraction of the corresponding DMSO vehicle values, these data represented net increases of approximately 5.0-, 11.8-, and 14.5-fold relative to untreated controls (Figure 6B).

Although DMSO alone produced a measurable elevation in MGO, consistent with its previously documented metabolic effects, IND markedly potentiated this response. At 300 μM, IND induced approximately five-fold greater MGO accumulation than its corresponding vehicle control, confirming that the increase is not attributable to solvent effects but rather to drug-specific metabolic disruption.

These findings provide direct evidence that enzymatic inhibition by IND translates into intracellular accumulation of a highly toxic glycolytic byproduct, consistent with a metabolic “bottleneck” scenario in which impaired TPI function promotes MGO generation while compromised GlAR activity limits detoxification capacity.

Consistent with elevated MGO levels, AGEs formation was dramatically enhanced following IND treatment. After correction for vehicle effects, trophozoites treated with 150, 215, and 300 μM IND exhibited net increases of 21.7-, 49.1-, and 84.47-fold over untreated controls (Figure 6C,D). At 300 μM IND, AGEs concentrations reached 5.96 μg/mL, compared with a basal level of 0.064 μg/mL in control cells.

Because MGO readily reacts with proteins to form AGEs, these data indicate that IND-induced carbonyl stress propagates into extensive secondary protein modification. The magnitude of AGEs accumulation suggests widespread glycation damage, likely impairing structural and enzymatic proteins essential to parasite viability.

### 2.7. IND Promotes Extensive Formation of MGO–Protein Adducts and Widespread Protein Carbonylation

**Figure 7:**
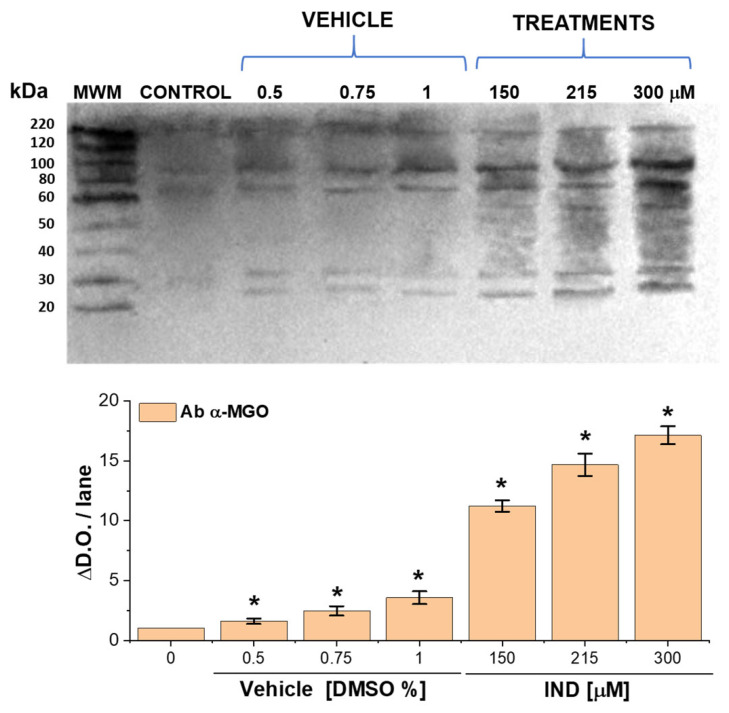
**IND enhances MGO-derived protein adduct formation in *Giardia lamblia* trophozoites.** Trophozoites were exposed for 72 h at 37 °C to IND (150, 215, and 300 μM) or to the corresponding DMSO vehicle controls (0.5%, 0.75%, and 1%, respectively, reflecting the final volume required for each IND concentration). Total protein extracts (200 μg per lane) were resolved by SDS–PAGE and transferred to PVDF membranes (0.2 μm pore size) using a semi-dry transfer system (20 V, 1 h). Membranes were incubated overnight with anti-MGO primary antibody (1:1000), followed by incubation with secondary antibody (1:2000). Immunoreactive bands were detected by enhanced chemiluminescence (luminol-based substrate) and documented using a Chemi-Doc RSC imaging system. The upper panel shows representative Western blotting detection of MGO-protein adducts in control, vehicle-treated, and IND-treated trophozoites. The lower panel presents normalized densitometric analysis, with the basal signal of the untreated control set to 1. Western blotting assays were performed. Data correspond to three independent biological replicates and are presented as the mean, with bars indicating experimental standard deviation. Statistical analysis was performed using one-way ANOVA followed by Tukey’s post hoc test, with statistical significance defined as *p* < 0.05 (* with respect to the corresponding control).

To determine whether the elevated intracellular MGO detected in IND-treated trophozoites resulted in direct chemical modification of parasite proteins, MGO-derived protein adducts were evaluated by immunodetection using anti-MGO antibodies (Figure 7). This strategy allowed us to move from quantification of soluble MGO to visualization of stable covalent adducts formed between MGO and cellular proteins.

Untreated trophozoites displayed a low but detectable basal signal of MGO–protein adducts. DMSO alone produced a concentration-dependent increase in immunoreactivity, with 1.6-, 2.46-, and 3.56-fold elevations at 0.5%, 0.75%, and 1% vehicle, respectively. These data confirm that the solvent exerts a measurable metabolic effect; however, the magnitude of this increase was modest compared with the response elicited by IND.

In contrast, trophozoites exposed to IND exhibited a dramatic accumulation of MGO-modified proteins (Supplementary Figure S3). Densitometric analysis revealed increases of 11.2-, 14.7-, and 17.1-fold relative to untreated controls at IND concentrations of 150, 215, and 300 μM, respectively. The scale of this effect greatly exceeded that observed with DMSO alone, demonstrating that the extensive protein modification is drug-specific rather than solvent-driven.

The broad distribution of immunoreactive bands indicates that multiple cellular proteins are modified, consistent with widespread protein carbonylation rather than selective targeting of a single substrate. These results demonstrate that accumulated MGO does not remain a soluble intermediate but instead reacts extensively with parasite proteins, forming stable adducts typically associated with functional impairment, misfolding, or degradation.

### 2.8. Flow Cytometric Evidence of Apoptotic-like Death in IND-Treated Trophozoites

Given that IND inhibits GlTPI and GlAR, leading to intracellular accumulation of MGO, enhanced AGEs formation, and widespread protein carbonylation, it was next evaluated whether this metabolic and redox imbalance culminates in parasite death. To characterize membrane alterations associated with cell death, Annexin V-FITC/propidium iodide (PI) staining followed by flow cytometry was performed.

A sequential gating strategy was applied in which trophozoites were first selected based on FSC/SSC parameters to exclude debris, and fluorescence quadrants were subsequently defined using unstained, Annexin V-only, and PI-only controls (Supplementary Figure S4A). These controls allowed empirical placement of thresholds to discriminate Annexin V and PI positivity and to define four operational populations: viable (Ann^−^/PI^−^; Q3), Annexin V-positive (Ann^+^/PI^−^; Q4), double-positive (Ann^+^/PI^+^; Q2), and PI-positive (Ann^−^/PI^+^; Q1). The necrosis control (H_2_O_2_-treated trophozoites) exhibited a heterogeneous distribution across Q1 and Q2, with enrichment in the double-positive quadrant, consistent with progressive and extensive membrane damage under oxidative stress (Supplementary Figure S4A).

In contrast, vehicle-treated cells (0.5–1% DMSO) showed a reproducible concentration-dependent increase in early apoptosis-like (Q4: 34–51%) and a minor increase in late apoptosis-like (Q2: 6–15%), with viable cells decreasing to 40–44%, strongly suggesting that the solvent itself induces measurable membrane perturbation and cellular stress.

IND treatment induced concentration-dependent changes in quadrant distribution. At 150 μM, the predominant population localized to the Annexin V-positive/PI-negative quadrant (Q4: 49%), with a concomitant decrease in viable cells (39%), suggesting early membrane remodeling characterized by phosphatidylserine exposure with largely preserved membrane integrity [19]. At 215 μM, a pronounced redistribution toward the double-positive quadrant was observed (Q2: 45%), accompanied by a reduction in the Annexin V-only population (Q4: 7%), consistent with progression from early membrane alterations to more advanced stages of membrane compromise. At 300 μM, the double-positive population remained dominant (Q2: 48%), while PI-positive/Annexin V-negative cells (Q1: 18%) increased, and viability further declined (16–22%). Notably, the overall distribution at this concentration closely resembled that of the necrosis control, indicating widespread and advanced membrane damage (Supplementary Figure S4B).

Across all conditions, the transition from Annexin V single-positive to double-positive and PI-positive populations occurred in a dose-dependent manner, reflecting a continuum of membrane alterations rather than discrete death categories. Accordingly, given the absence of canonical apoptotic machinery in *G. lamblia*, these populations are interpreted conservatively as Annexin V/PI-defined phenotypes indicative of progressive membrane remodeling and loss of integrity, rather than definitive evidence of classical apoptosis.

**Figure 8:**
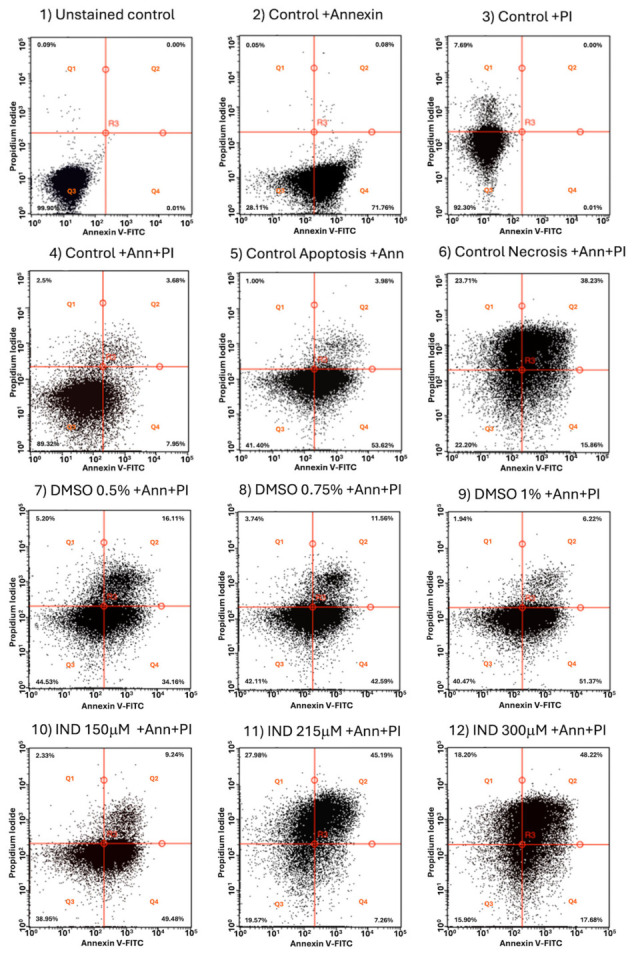
**Flow cytometric analysis of apoptosis-like and necrosis in *Giardia lamblia* trophozoites following IND treatment.** Trophozoites were incubated for 72 h under the indicated conditions and stained with Annexin V-FITC and PI to discriminate viable and dying cell populations. A total of 30,000 events per sample were analyzed. Upper panels: Controls used to validate gating and quadrant discrimination. (**1**) Untreated trophozoites; (**2**) Annexin V-FITC staining only; (**3**) PI staining only; (**4**) dual Annexin V/PI staining control; (**5**) apoptosis control, cells treated with H_2_O_2_ (20 µM, 16 h) + Annexin V staining; (**6**) necrosis control, cells treated with H_2_O_2_ (320 µM, 16 h) stained with Annexin V/PI staining. Vehicle controls: (**7**–**9**) Trophozoites treated with DMSO at 0.5%, 0.75%, and 1%, corresponding to the final concentrations required for 150, 215, and 300 µM IND, respectively. Quadrant distribution: viable cells (Ann^−^/PI^−^; Q3), early apoptosis-like (Ann^+^/PI^−^; Q4), late apoptosis-like (Ann^+^/PI^+^; Q2), and necrosis (Ann^−^/PI^+^; Q1). Untreated trophozoites maintained high viability with minimal apoptotic or necrotic populations. DMSO-treated cells exhibited a moderate, concentration-dependent increase in apoptosis-like, consistent with vehicle-induced cellular stress. Data are representative of two independent biological experiments.

Overall, these results strongly suggest that IND induces a concentration-dependent shift from viable cells to Annexin V-positive and subsequently double-positive/PI-positive populations, consistent with escalating cellular damage and loss of membrane integrity (Figure 8; Supplementary Figure S4B, Table S2).

### 2.9. IND Induces Drastic Ultrastructural Disruption in Giardia lamblia Trophozoites

Having demonstrated that IND inhibits GlTPI and GlAR, promotes MGO accumulation, enhances AGEs formation, induces widespread protein carbonylation, and triggers apoptotic-like death, we next investigated whether these biochemical alterations translate into structural damage at the ultrastructural level.

Transmission electron microscopy (TEM) was performed to evaluate the integrity of organelles and cytoskeletal structures, with particular attention to the adhesive disc and microtubule (MT) system, key protein assemblies required for trophozoite morphology, attachment to the intestinal epithelium, and cytokinesis.

**Figure 9:**
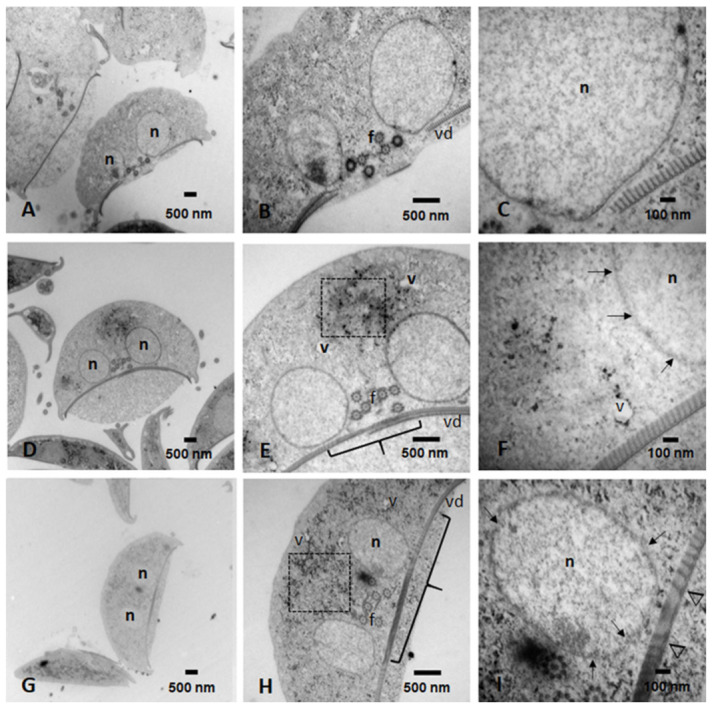
**Ultrastructural alterations induced by IND in *Giardia lamblia* trophozoites.** Transmission electron microscopy of untreated control cells (**A**–**C**), trophozoites incubated for 72 h with 215 μM IND (**D**–**F**), and 300 μM IND (**G**–**I**). Control trophozoites (**A**–**C**) exhibit preserved nuclear and plasma membrane integrity, homogeneous cytoplasmic electron density, intact ventral (adhesive) disc, and well-organized cytoskeletal structures. Cells treated with 215 μM IND (**D**–**F**) show early ultrastructural alterations, including reduced cytoplasmic electron density and electron-dense precipitates (boxed areas) in the cytoplasm, frequently associated with vacuoles (**V**). Disruption of the nuclear membrane (small arrows) and partial disorganization of the adhesive disc (brackets; arrowheads) are evident. At 300 μM IND (**G**–**I**), trophozoites display severe structural damage, including fragmentation of the nuclear envelope, extensive destruction of the ventral disc (vd), and marked cytoskeletal disorganization. Basal body and flagellar (f) alterations are also observed. Nucleus (*n*); vacuole (V); ventral disc (vd); flagella (f).

Untreated control cells displayed the characteristic ultrastructural features of healthy *G. lamblia* trophozoites (Figure 9A–C). Nuclear and plasma membranes were intact, the cytoplasm exhibited homogeneous electron density consistent with abundant glycogen granules and ribosomes, and peripheral vacuoles and endoplasmic reticulum cisternae were clearly distinguishable. The lateral crest and adhesive disc were well defined, and disc-associated MT arrays were orderly and compact. These findings confirm the preservation of cellular architecture and metabolic competence, establishing the structural “gold standard” for comparison with IND-treated cells.

To visualize the greatest effect on trophozoite ultrastructural damage, the two highest concentrations of IND were explored. Trophozoites treated with 215 μM exhibited clear but progressive ultrastructural alterations (Figure 9D–F). Cells displayed mild swelling and reduced cytoplasmic electron density, suggesting metabolic imbalance and partial loss of intracellular organization. Electron-dense precipitate clusters were observed within the cytoplasm and vacuolar compartments, compatible with protein aggregation and/or accumulation of carbonyl-modified material, in agreement with the elevated MGO levels, AGEs formation, and MGO–protein adducts previously described.

Structural deterioration was also evident at the nuclear and cytoskeletal levels. Fragmentation of the nuclear envelope was detected, along with partial disruption of the adhesive disc and alterations in the central pair of MTs (Figure 9E). In some cells, the adhesive disc remained partially preserved but exhibited reduced electron density and compaction of its protein components (Figure 9F), consistent with progressive cytoskeletal destabilization. These changes indicate that metabolic and carbonyl stress are already impacting structural protein assemblies essential for parasite viability.

At 300 μM IND, ultrastructural damage was markedly exacerbated (Figure 9G–I). Trophozoites exhibited reduced cell volume—consistent with advanced cell death—along with extensive nuclear membrane fragmentation. Severe destruction of the adhesive disc was evident, with large areas of structural discontinuity and pronounced disorganization of disc-associated MT arrays. Basal bodies appeared extensively damaged, and MT integrity was profoundly compromised. Because the adhesive disc and basal bodies depend on a stable MT scaffold, their disruption represents functional annihilation of the parasite’s structural framework.

Importantly, these ultrastructural findings correlate with the severe loss of viability and the shift toward late apoptosis-like and necrosis detected by flow cytometry, as well as with the extensive protein carbonylation revealed by immunodetection. The observed MT alterations are also consistent with previous reports indicating that IND binds tubulin and interferes with MT polymerization dynamics, leading to mitotic arrest and cytoskeletal disruption in mammalian cells [20].

In *G. lamblia*, where the adhesive disc and basal bodies critically depend on a stable MT network, perturbations in MT assembly are likely to directly contribute to the structural collapse observed here.

Moreover, aldose reductase activity has been reported to be altered by its association with intact MTs [21]. Thus, MT disorganization in IND-treated trophozoites may further compromise GlAR function, amplifying redox imbalance and reinforcing carbonyl stress. This potential feedback loop, in which cytoskeletal disruption exacerbates metabolic dysfunction, adds a mechanistic layer linking structural damage to biochemical collapse.

Finally, the pattern of adhesive disc and basal body disruption resembles ultrastructural alterations previously reported in trophozoites treated with the antineoplastic compound casiopein [22], suggesting that cytoskeletal collapse may represent a common terminal outcome of severe metabolic and oxidative stress in *G. lamblia*.

Taken together, TEM analysis integrates structural evidence with biochemical and cytometric data, supporting a multifactorial mechanism of IND action in *G. lamblia* that involves coordinated metabolic disruption, carbonyl stress, microtubule destabilization, and irreversible cellular damage culminating in parasite death.

### 2.10. Comparative Structural Docking Reveals Preferential Binding of IND to Giardial Enzymes

**Figure 10:**
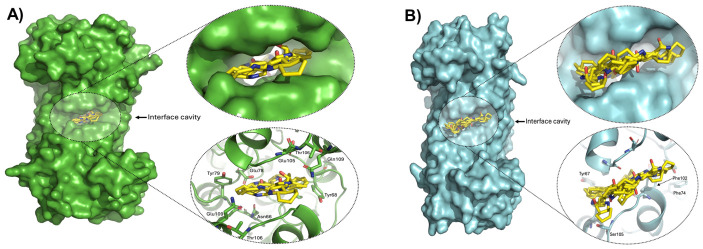
**Molecular docking analysis of IND at the dimer interface of crystallographic structures of GlTPI and HsTPI.** (**A**) Surface representation of the crystallographic structure of GlTPI (PDB: 4BI6) shown as a homodimer (green), illustrating the preferred docking poses of IND localized within the interfacial cavity. The upper oval inset shows a zoomed view of IND positioned inside the cavity, indicating deep ligand burial. The lower oval inset highlights the neighboring interfacial residues contributing to ligand stabilization through hydrophobic and aromatic interactions. (**B**) Surface representation of the crystallographic structure of HsTPI (PDB: 2JK2) shown as a homodimer (cyan) with the corresponding docked poses of IND at the dimer interface. Oval insets display the reduced cavity dimensions (upper inset) and the surrounding amino acid environment (lower inset), revealing a more superficial accommodation of IND and fewer stabilizing contacts compared with the Giardial enzyme. Docked conformers of IND are shown as stick representations, emphasizing structural differences in cavity size and residue composition between parasite and human TPI interfaces. Figures modeled with PyMOL, Molecular Graphics System v2.5.0 (Schrödinger, Inc., New York City, NY, USA).

Docking simulations revealed that IND preferentially targets the dimer interface of the GlTPI crystallographic structure (Figure 10A). The most favorable binding pose reached −8.2 kcal/mol, representing the lowest energy among all evaluated cavities. Importantly, several high-scoring poses (−7.3 to −6.3 kcal/mol) consistently clustered within the same interfacial region, indicating structural convergence toward this cavity as the principal binding site. Structural inspection showed that IND inserts deeply into the interface cavity of GlTPI (Figure 10A), where their stabilization is primarily mediated by polar and hydrogen-bonding interactions involving residues Tyr68 (chains A and B), Thr106 (chains A and B), Asn66 (chain A), Glu78 (chain A), and Gln109 (chains A and B). The presence of these residues strongly suggests that hydrogen bonding and polar contacts may play a dominant role in ligand stabilization, allowing IND to be extensively docked across the dimer interface.

By contrast, docking to HsTPI yielded less favorable binding energies, with a best overall value of −6.0 kcal/mol and interfacial docking reaching only −5.8 kcal/mol (Figure 10B). Ligand poses in HsTPI were markedly more superficial, showing reduced burial and fewer stabilizing contacts. The smaller cavity volume and residue composition—Tyr67, Phe102, Phe74, and Ser105 (all in chain A), limit optimal packing and geometric complementarity. Although aromatic residues are present, their spatial arrangement appears insufficient to promote effective π-stacking and deep ligand docking, consistent with the weaker docking energies observed.

An important structural feature to consider is the volume of the internal interfacial cavity in both enzymes. In GlTPI, this cavity measures 394.75 Å^3^, more than double that of its human counterpart (161.50 Å^3^). This substantial difference suggests that IND can be accommodated more deeply within the parasitic enzyme, increasing the potential contact surface area and providing a structural basis for the observed differences in binding energies.

To determine whether IND directly targets GlAR and to evaluate its selectivity relative to HsAR, molecular docking simulations were performed focusing on predicted ligand-binding pockets, particularly the NADP(H)-binding cleft, which is essential for catalytic activity.

**Figure 11:**
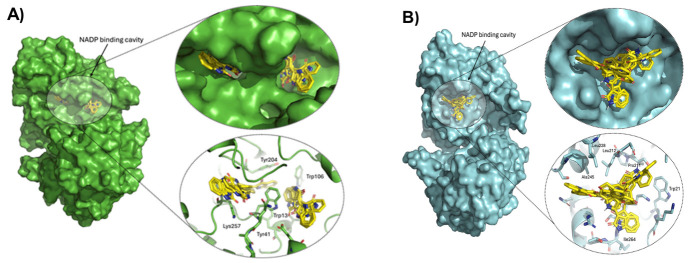
**Molecular docking analysis of IND at the NADP(H)-binding cleft of crystallographic structures of GlAR and HsAR.** (**A**) Surface representation of the crystallographic structure of GlAR (PDB: 3KRB) showing the preferred docking poses of IND localized within the predicted NADP(H)-binding pocket. Docked conformers (stick representation) cluster within the cofactor-binding cleft. The upper oval inset shows a magnified view of IND positioned inside the cavity, indicating deep ligand accommodation. The lower oval inset highlights the principal neighboring residues, including aromatic residues (Trp and Tyr), forming stabilizing π–π interactions with the indole rings of IND. (**B**) Surface representation of the crystallographic structure of HsAR (PDB: 2ALR) with the corresponding docked poses of IND mapped to the NADP(H)-binding region. Oval insets illustrate the surrounding amino acid environment (including Trp, Leu, Pro, and Ala), revealing reduced aromatic complementarity and a more superficial mode of ligand stabilization compared with the parasite enzyme. Figures modeled with PyMOL, Molecular Graphics System v2.5.0 (Schrödinger, Inc., New York City, NY, USA).

In the GlAR crystallographic structure, the NADP(H)-binding pocket was identified as the highest-probability cavity. All nine top-ranked docking poses of IND localized within this region (Figure 11A), demonstrating strong preferential binding. The most favorable pose reached −9.38 kcal/mol, while the remaining conformations ranged from −9.27 to −8.58 kcal/mol, indicating highly consistent and stable interactions within the same structural pocket. Structural analysis shows that IND occupies the NADP(H)-binding cleft of GlAR (Figure 11A), stabilized by a network of aromatic and polar interactions with Tyr204, Trp106, Tyr41, Trp13, and Lys257. The aromatic residues likely engage in π–π stacking with the indole rings of IND, while Lys257 provides further stabilization through polar contacts. Collectively, these interactions anchor the ligand firmly within the cofactor-binding site. The positioning of IND within this cleft is structurally compatible with competitive interference with NADP(H)-binding, providing a direct structural explanation for the experimentally observed reduction in GlARr activity.

In contrast, docking to the HsAR structure also localized IND within the NADP(H)-binding region; however, binding affinities were substantially weaker (Figure 11B). The best-ranked pose had an energy of −6.31 kcal/mol, with the remaining conformers showing similarly less favorable energies. The interaction network in HsAR comprises residues such as Trp21, Leu212, Leu228, Pro211, Ile264, and Ala245, which offer limited opportunities for optimal aromatic stacking. The prevalence of aliphatic and non-aromatic residues reduces geometric complementarity to IND; although Trp21 may facilitate π-interactions, the lack of a robust aromatic environment results in diminished ligand stabilization compared to GlAR. Consequently, ligand stabilization was markedly weaker than in GlAR.

## 3. Discussion

The frequency of therapeutic failure in giardiasis represents an emerging clinical concern. Resistance to metronidazole, the long-standing first-line treatment, has been reported in up to 20% of cases, with recurrence rates approaching 90%, indicating persistent parasite adaptation. Resistant *G. lamblia* isolates have been reproducibly characterized, and cross-resistance to other nitroimidazoles such as tinidazole has also been documented. In addition, experimental studies show that resistance to one drug can facilitate adaptation to others; for example, furazolidone-resistant strains more readily acquire resistance to quinacrine, and albendazole resistance develops faster in resistant backgrounds, leading to a multidrug-resistant (MDR) phenotype [23]. This resistance landscape underscores the need to identify new therapeutic strategies that target essential metabolic pathways less susceptible to classical resistance mechanisms. Thus, structural inhibition of relevant enzymes involved in energy metabolism and detoxification, such as GlTPI and GlAR, represents a rational strategy, particularly because glycolysis is indispensable for *Giardia* survival. The present study, therefore, explores the structural basis for IND-mediated inhibition as a selective, non-redox-dependent strategy that may help overcome current limitations in giardiasis chemotherapy.

In this context, the pharmacological versatility of IND becomes particularly relevant. Natural and repurposed compounds with multi-target capacity, particularly those investigated in traditional medicine, have emerged as valuable source of novel therapeutic strategies [24]. In mammalian systems, IND has been associated with cytoprotective effects, including the inhibition of apoptosis in retinal ganglion cells through alteration of the PI3K/AKT signaling pathway [25,26]. Interestingly, our results reveal a mechanistically contrasting behavior in *G. lamblia*. Rather than activating survival pathways, IND acts as a direct metabolic disruptor, producing dose-dependent cytostatic and cytotoxic effects on trophozoites. After correcting for solvent-associated effects, IND maintained clear dose-dependent inhibition of both enzymes, strongly suggesting a pharmacological action.

Importantly, our results also revealed measurable effects of the vehicle (DMSO), consistent with its known biochemical properties, including membrane interference, transient pore formation and protein conformational effects [27,28]. Although DMSO induces measurable biological stress in *Giardia*, its effects remained consistent across experimental conditions. Notably, the most pronounced biological and enzymatic alterations were observed at higher IND concentrations, clearly exceeding those produced by the vehicle alone. These observations reinforce that, while IND can act as a cell-protective signaling factor in mammalian models, in *Giardia* it appears to function primarily through structural enzymatic interference, simultaneously altering glycolysis and oxidative defense systems. However, within the high concentration range employed, the observed phenotype may partially arise from the combined contribution of on-target and off-target interactions. IND and its derivatives are well established as ATP-competitive inhibitors of glycogen synthase kinase 3 and cyclin-dependent kinases (GSK/CDK) [12], where they interact with conserved structural features of the kinase catalytic cleft and stabilize inactive conformations. This dual activity underlies many of the cellular effects attributed to IND in mammalian systems, particularly those related to cell cycle regulation and signaling. In *G. lamblia,* however, canonical GSK/CDK homologs are either structurally divergent or functionally adapted, raising the possibility that IND may preferentially interact with alternative proteins that retain partial conservation of the ATP-binding pocket or surrounding regulatory elements. Such targets could include *Giardia*-specific kinases or other nucleotide-binding enzymes that mimic important aspects of GSK/CDK architecture, thereby permitting IND accommodation despite sequence divergence. Consequently, the phenotypic effects observed in *Giardia* are likely to reflect not only modulation of putative kinase homologs but also engagement of structurally related off-target proteins, consistent with a broader and potentially parasite-selective interaction profile.

Our proposed dual metabolic targeting provides mechanistic explanation for trophozoite death and underscores the importance of pharmacological strategies against parasites with emerging multidrug resistance.

Importantly, while this dual metabolic targeting provides a coherent mechanistic framework for trophozoite death, we recognize that the linkage between enzyme inhibition, MGO accumulation, and downstream cytotoxicity is supported by convergent evidence rather than direct causal demonstration. In addition, we cannot exclude that parallel interactions with other, non-characterized targets may contribute to the amplification of carbonyl stress and cellular damage under these conditions.

Our findings demonstrate that IND exerts a dose- and time-dependent antiproliferative effect on *G. lamblia* trophozoites, consistent with the progressive inhibition of native GlAR activity. While this dual metabolic targeting provides a coherent mechanistic framework, we recognize that the linkage between enzyme inhibition, MGO accumulation, and downstream cytotoxicity is supported by convergent evidence rather than direct causal demonstration. Importantly, although the vehicle produced measurable proteostatic effects, the inhibitory action of IND was clearly stronger, confirming a direct pharmacological contribution beyond solvent-associated effects. These observations help clarify previous reports of marginal proliferation increases at very low IND concentrations [14]. By incorporating appropriate untreated and vehicle controls, our study refines this interpretation and supports that IND produces a genuine antiproliferative effect linked to metabolic interference.

At the level of native enzyme activity, DMSO increased the apparent catalytic activity of both enzymes relative to untreated controls, consistent with the known ability of organic solvents to induce reversible conformational changes [29]. Residual activities from IND-treated trophozoites were therefore normalized against DMSO-only controls to separate solvent effects from compound-specific actions. After correction, IND exhibited a concentration-dependent biphasic effect: lower concentrations produced a relative increase in residual activity (possibly reflecting transient metabolic adaptation), while higher concentrations shifted toward clear inhibition, indicating progressive enzymatic inhibition. This transition supports GlTPI and GlAR as relevant molecular targets contributing to IND’s antiparasitic action.

Meanwhile, at the level of recombinant enzymes, the differential sensitivity of parasitic and human targets was further confirmed. GlTPIr showed a biphasic response: short-term exposure increased activity, while prolonged incubation led to a clear decline, suggesting progressive structural perturbation. HsTPIr remained minimally affected even at substantially higher IND concentrations, indicating marked selectivity toward the parasite enzyme. In contrast, GlARr showed early sensitivity to IND, with inhibition evident at short exposure times. HsAKR1-A1r displayed delayed but pronounced sensitivity upon prolonged incubation, suggesting time-dependent off-target effects on this host enzyme. Thus, IND shows favorable selectivity for parasite TPI and AR, but prolonged exposure may increase off-target interactions with the human AKR enzyme [21].

One of the major effects previously described for omeprazole treatment in *Giardia* was a substantial increase in MGO formation and AGEs via GlTPI impairment [8]. Our findings extend this concept: the native detoxification capacity mediated by GlAR is insufficient to counteract IND-induced carbonyl overload. Notably, IND produced markedly stronger accumulation of glycation products at substantially lower concentrations than omeprazole, highlighting superior potency in triggering metabolic intoxication.

As a corollary, we propose a mechanistic model in which IND-induced dual inhibition of GlTPI and GlAR promotes MGO accumulation and subsequent cell death. This idea is supported by convergent evidence: (i) direct inhibition of recombinant enzymes, (ii) a concentration-dependent rise in intracellular MGO (up to 14-fold), (iii) concomitant increases in AGEs (up to 84-fold) and MGO–protein adducts (up to 17-fold), and (iv) consistent downstream damage, including protein carbonylation, ultrastructural alterations, and apoptosis-like death. Although causality was not formally established (e.g., via rescue experiments), these findings support a strong mechanistic association rather than definitive proof. This interpretation is strengthened by Giardia’s lack of a canonical glyoxalase system and its reliance on the polyol pathway for MGO removal [30]. The concurrent disruption of glycolytic substrate flux (via GlTPI) and detoxification capacity (via GlAR) creates a negative metabolic synergy that likely underlies the pronounced accumulation of AGEs and their resulting cytotoxic effects.

Flow cytometry analysis demonstrated that IND induces an apoptotic-like response in *Giardia* trophozoites, functionally linking metabolic alterations with programmed cell death. The cytometric profiles, together with biochemical evidence of protein modification and carbonyl stress, reinforce that IND triggers metabolic intoxication as a central mechanism of parasite elimination. However, further studies, such as genetic or pharmacological modulation of MGO levels, will be required to dissect the causal contribution of MGO in greater detail.

Comparable cytometric patterns have been reported for other giardicidal compounds, including garcinol [31] and ivermectin [32], although upstream triggers differ. Notably, linearolactone was also predicted to target an aldose reductase homolog [10], suggesting that interference with carbonyl detoxification may represent a broader vulnerability in *Giardia* metabolism. From a chemical standpoint, thiol, amino, and guanidino groups are highly reactive toward MGO [33,34,35,36]. This is especially relevant in *Giardia*, which lacks classical oxidative stress enzymes and depends largely on cysteine-based buffering systems [37]. IND-induced MGO accumulation likely enhances modification of cysteine-containing proteins, weakening the parasite’s protective mechanisms and promoting apoptosis-like death.

In silico docking of IND with GlTPI and GlAR yielded comparative profiles consistent with prior structural analyses, supporting the interfacial and cofactor-binding regions as plausible pharmacological hotspots. In GlTPI, preferential localization at the dimer interface aligns with studies showing that hydrophobic packing critically affects dimer stability and catalytic efficiency [38]. Similar interfacial docking has been described in *Leishmania mexicana* TPI [39], supporting that interface-directed ligands may disrupt functional dimer stability. The larger interfacial cavity in GlTPI relative to HsTPI provides a structural rationale for selective ligand accommodation. For GlAR, docking results are consistent with studies showing that aromatic residues (Trp, Tyr) stabilize inhibitors within the cofactor-binding pocket [40,41]. Docking studies with linearolactone also identified GlAR as a potential target [10]. From a chemical perspective, the indole scaffold of IND may contribute to π-stacking interactions with Trp/Tyr residues, enhancing docking stability [42]. However, because our analysis relied on automated docking only, the proposed binding modes and selectivity claims should be interpreted as supportive rather than definitive. Experimental validation through X-ray crystallography or site-directed mutagenesis would be required to confirm precise binding interactions.

The present findings align with recent advances in natural product-based drug discovery, emphasizing multi-target mechanisms. Piperlongumine and piperine analogues inhibit thioredoxin reductase, increase reactive oxygen species, and modulate p38 and AKT/mTOR signaling pathways, thereby exerting antiproliferative effects against drug-resistant cancer cells [43]. Similarly, IND in *Giardia* disrupts two essential nodes, glycolysis (GlTPI) and carbonyl detoxification (GlAR), producing synergistic metabolic damage leading to apoptosis-like death. This dual-target strategy mirrors the rationale behind multi-target kinase and HDAC inhibitors in cancer therapy, where simultaneous modulation of multiple pathways overcomes resistance and enhances efficacy [44]. Furthermore, the metabolic disruption induced by IND in *Giardia* bears conceptual similarities to lipid-mediated reprogramming in cancer biology, where altered metabolic pathways influence stress responses, cell demise, and intercellular crosstalk within the tumor microenvironment [45]. In *Giardia*, the accumulation of MGO and AGEs following IND treatment creates a sustained carbonyl stress environment that impairs thiol-dependent defenses and triggers programmed cell death. Thus, targeting structurally divergent yet metabolically essential hubs, such as the dimer interface of TPI or the cofactor-binding cleft of AR, represents a robust strategy for developing selective anti-infective agents that bypass classical resistance mechanisms, reinforcing the value of integrating biochemical validation with natural product-inspired drug design.

Further studies in murine models are required to validate the in vivo efficacy of IND and substantiate its proposed protein targets.

## 4. Materials and Methods

### 4.1. Reagents and General Materials

The following reagents and materials were used for the experimental procedures of this study. IND was purchased from SIGMA-Aldrich (St. Louis, MO, USA), Cat.No. PHL89716-10MG. All other reagents were of analytical grade and purchased from Sigma-Aldrich suppliers. Methylglyoxal (MGO), CatNo. M0252-25ML; Phosphate-buffered saline (PBS), Cat.No. P2272-500ML; Indirubin (IND) Cat.No. PHL89716-10MG. Bicinchoninic acid reagent, Cat.No. D8284-10G. Ammonium sulphate (NH_4_)_2_SO_4_) Cat.No. A4915-1KG. Ethylenediaminetetraacetic acid (EDTA) Cat.No. 798681-1KG. Glycerol-3-Phosphate Dehydrogenase (GDH) Cat.No. 10127752001. DL-Ditiotreitiol (DTT) Cat.No. D0632-5G. Ampicillin, Cat.No. A9518-5G. Ceftazidime hydrate Cat.No. 6987-1G. Trypan blue 4 g/L, Cat.No. T8154-20ML. 8-Anilino-1-naphthalenesulfonic acid (ANSA) Cat.No. A1028-100G. Tween 20, Cat.No. P1379-25ML; Sodium dodecyl sulfate, (SDS) Cat.No. L4390-500G. Glutaraldehyde solution, Cat.No. 340855-25ML. Polyvinylidene Difluoride membranes (PVDF Amersham™Western blotting membrane Hybond^®^P, PVDF 0.22 μm, Cat.No. GE10600021; 3,3′,5,5′-tetramethylbenzidine TMB Cat.No. T2885-5G. Trizma-base, Cat.No. T1503-500G. Nicotinamide adenine dinucleotide reduced (NADH) (MERCK, Darmstadt, Germany, Cat.No. N1161). Dihydronicotinamide adenine dinucleotide phosphate tetrasodium salt (NADPH) Roche Cat.No. 10107824001, Germany. TYI-S-33 media, fetal bovine serum (FBS), were acquired from Gibco (Thermo Fisher Scientific, Waltham, MA, USA or Biowest, Cat.No. S162C-500). Dimethylsulfoxide (DMSO) ATCC, 4-X-5. 10,801 Virginia, EE. UU., University Blvd. Manassas, Va 20,110 703-365-2700. LB broth (Thermo-Fisher Scientific, Cat.No. 12780029). Profinity™ IMAC Resin (immobilized metal affinity column), Ni-charged, 10 mL, CatNo. 1560131. Amicon^®^ Ultra Centrifugal Filters MWCO 30 kDa Merck-Millipore, Cat.No. UFC9030. MagicMark™ XP Western Protein Standard (ThermoFisher Scientific, Waltham, MA, USA, Cat.No. LC5602); absolute methanol (J.T. Baker) grade HPLC, Capitol Scientific, Austin, TX, USA, Cat. No. 9093-03. Glycine, MP Biomedicals, Solon, OH, USA, Cat.No. 194825; 340855-25ML. Horizontal transfer chamber (Bio-Rad, Hercules, CA, USA, Trans-Blot^®^ SD Semi-Dry Electrophoretic Transfer Cell Cat.No. 170-3940. NaCl VWR, Leicestershire, UK, Cat.No. 0241-2.5KG. Ab anti-methylglyoxal (α-MGO) (ABCAM, Waltham, MA, USA [9F11] ab243074). Ab anti-MGO (α-MGO), SantaCruz H11, Dallas, TX, USA, Cat. No. sc-166785 HRP. Ab peroxidase-bound anti-mouse IgG, Cell Signaling HRP-linked, Danvers, MA, USA, Cat.No. 7076s. Luminol Santacruz Biotec (ImmunoCruz Western Blotting Luminol Reagent, Cat.No. sc-2048, Santacruz Biotechnology, Inc., Dallas, TX, USA. Transilluminator Bio-Rad, ChemiDoc XRS+ Gel Imaging System. Cary 50 spectrophotometer, Agilent Technologies, Santa Clara, CA, USA. Perkin-Elmer LS-55 spectrofluorometer, PerkinElmer Life and Analytical Sciences 710 Bridgeport Avenue Shelton, CT 06484-4794 USA. Microplate spectrophotometer, Epoch, BioTek, Winooski, VT, USA. AGEs ELISA kit MyBioSource, San Diego, CA, USA. Guava^®^ easyCyte™ flow cytometer Cytek^®^ Biosciences, Fremont, CA, USA. Data were acquired and processed using InCyte™ Software v3.1, Merck Millipore. BRANSON Digital Sonifier 450 cell disruptor, Danbury, Connecticut, U.S.A. Annexin V-FITC/Propidium Iodide apoptosis detection kits were obtained from Biolegend (San Diego, CA, USA). PyMOL v2.5.0 software for structural analyses was provided by Schrödinger Inc., New York, NY, USA.

### 4.2. Giardia Culture

*G. lamblia* WB strain was previously acquired from the American Type Culture Collection (ATCC), cultured, harvested, and maintained in medium TYI-S-33 supplemented with 10% heat-inactivated bovine serum as previously described [46]. Metronidazole resistance was induced in vitro in WB strain (23.37 mM or 4 mg/mL) using conventional methods previously described in [47].

### 4.3. Indirubin Treatment in Giardia lamblia Trophozoites

Briefly, treatments of 5 × 10^4^ trophozoites in 7 mL of medium TYI-S-33 supplemented with 10% fetal calf serum and antibiotics Ampicillin and Ceftazidime at 0.5 mg/mL for 72 h at 0, 150, 215, and 300 μM-IND were incubated and grown, containing a maximal concentration of vehicle-DMSO 1%. At the end of the experiments, cells were chilled and concentrated by centrifugation at 3800 rpm for 12 min. Subsequently, the number of trophozoites and their viability were determined by the trypan blue exclusion method.

One fraction of each tube was used to obtain cellular extracts at a concentration of 5 × 10^6^ trophozoites/0.1 mL in solution buffered with phosphate (PBS), clarified by centrifugation at 12,000 rpm for 20 min. At 4 °C, the other fraction was used to assess the residual activity of native enzymes triosephosphate isomerase and aldose reductase, and to carry out assays for MGO and AGEs, Western Blotting, and Flow cytometry (CFS), respectively.

### 4.4. Purification of Recombinant Enzymes

Recombinant enzymes, HsTPIr and GlTPIr, were purified as described in [48,49], respectively. Purification of recombinant AKR enzymes was carried out with some modifications of methods reported by HsAKR1-A1 (HsAR) [50,51], respectively; each one of the human AKR and Giardial (GlAR) enzymes was expressed in competent *E. coli* BL21-DE3pLysS, transformed by the heat-shock plasmid pET3a containing the genes of GlAR or HsAKR1-A1 (genes were synthesized by Gene Universal (Newark, DE, USA) and GenScript (Piscataway, NJ, USA), respectively). These plasmid construction genes contained at the N-terminus an additional six histidine residues (6xHis-tag) and a tobacco Etch Virus Protease (ETVp) cleavage site. These colonies were subsequently selected for antibiotic resistance and grown in LB (Luria–Bertani) medium at 37 °C until an optical density of 1.0 at 600 nm was reached. The recombinant enzymes were obtained from bacterial lysates of competent *E. coli* BL21-DE3pLysS.

The cultures were previously induced with 0.4 mM IPTG and grown overnight at 30 °C. Harvested cells were concentrated by centrifugation at 6000 rpm for 12 min at 10 °C. Finally, AKR enzymes were prepared from bacterial pellets obtained from an equivalent of either 2 Lt of culture by ultrasonication in a cell disruptor. These cells were resuspended in 40 mL of lysis buffer, NaCl 0.3 M, Tris-HCl 50 mM, pH 8.0, PMSF 0.1% (*v*/*v*) plus imidazole 10 mM, and sonicated seven times, each one for 45 sec. and arrested at 1:45 in an ice water bath. Debris was removed by centrifugation (9000 rpm, 30 min at 10 °C). The supernatant was applied to the column IMAC, incubated for 30 min on ice batch under agitation with 10 mL of resin previously equilibrated in lysis buffer; the protein batch was eluted with Imidazole 250 mM. To remove nonspecifically bound material, the column was washed with five volumes of lysis buffer.

Finally, AKR enzymes were eluted from IMAC with 20 mL of lysis buffer containing imidazole 250 mM. After the addition of glycerol to a final concentration of 20% (*v*/*v*), the AKR enzymes were stored at −20 °C in glycerol 20% until required for experimental IND inhibition. The AKR enzymes without NTer-6xHistag were subsequently subjected to ETVp7M (Etch Tobacco Virus Protease 7M) in buffer containing Tris 50 mM, pH 8.0, EDTA 0.5 mM, and DTT 1 mM to obtain AKR vs. ETVp using a protease/substrate ratio of 1:60, incubated overnight at 25 °C. Digestion reactions were performed by incubating Histag-AKR1-A1 and Histag-GlAR + His7-TEVp7M as described in [52]. And then they were recovered from the digest in batches on an IMAC column. After washing the pre-equilibrated IMAC column (80 mL of buffer NaCl 300 mM, Tris-HCl 100 mM, pH 8.0), the AKR proteins eluted were concentrated by centrifugation at 4500 rpm in an MWC 30 kDa centricons ultrafiltration at 10 °C and pooled and stored at 4 °C until use in an elution buffer [50] plus glycerol 20%.

### 4.5. Residual Activity of Native and Recombinant Enzymes Giardial Triosephosphate Isomerase and Human Triosephosphate Isomerase

To confirm the effect of IND-treatment on residual activity of native enzymes, (GlTPI) controls and treated trophozoites (Control, vehicle-DMSO 1% and 150, 215, 300 μM IND) were washed three times with ice-cold PBS pH 7.4 and lysed by alternating freeze–thaw cycles for 1 min, followed by heating to 37 °C for 45 s and incubation for 15 s in liquid nitrogen. Afterwards, the lysates were centrifuged at 12,000 rpm for 20 min at 4 °C to obtain a clarified lysate. Protein concentrations were determined using the bicinchoninic acid assay. The enzymatic activities of the native and recombinant TPI enzymes (GlTPI and HsTPI, respectively) were determined spectrophotometrically using a Cary 50 spectrophotometer under conditions optimized for each enzyme.

Activities were measured by monitoring the formation of NAD at 340 nm; their residual enzymatic activities were determined using a coupled system involving the disappearance of NADH at 340 nm, which reflects the enzyme-catalyzed conversion of the substrate glyceraldehyde 3-Phosphate (G3P) to dihydroxyacetone phosphate (DHAP), coupled to α-GDH enzyme. Reaction mixtures were prepared at 0.6 mL and loaded in quartz cuvettes containing trietanolamine 100 mM buffer (TE) at pH 7.4, NADH 2 mM, G3P 1 mM, and α-GDH 0.9 U. To determine the residual activity in native enzyme GlTPI, in the reaction mix, 2 μL of cytosolic cellular extract at (5 × 10^7^ trophozoites/mL) were added, previously sonicated and clarified to allow formation of NAD [48].

To determine residual activity in experiments with both recombinant enzymes, in the case of GlTPIr, samples included a control and DMSO vehicle, with 1% representing the highest volume of IND at 150, 215, 300, and 450 µM incubated for 2 h and 24 h.

For HsTPIr, residual activity readings were obtained with 0, 50, 100, 250, 500, and 1000 µM IND, incubated for 24 h at 37 °C. Residual activity was monitored with the enzyme at 5 ng/mL, previously incubated at 0.2 mg/mL. The spectrophotometric disappearance of NADH at 340 nm was observed in the reaction mixture containing α-GDH as the coupling enzyme in TE buffer. The normalized residual activity was set as the control (100%). The decrease in absorbance ε340 = 6.22 mM^−1^ cm^−1^ was recorded over the first three min. at 25 °C, and the enzyme activity was calculated as the rate of NAD formation (ΔAbs340/min) [48].

### 4.6. Residual Activity of Native and Recombinant Aldose Reductase Enzymes

Native Aldose reductase activity of *Giardia* (GlAR) was assessed as described in [53] with some modifications. For GlAR native, the reactions for residual activity determinations in a reaction mix of 500 µL containing MGO 1.75 mM as a substrate and NADP(H) 1 mM in NaH_2_PO_4_ 100 mM, pH 7.0, were initiated by total protein extract from trophozoites, adding 20 μL at 5 × 10^6^ trophozoites/100 μL of extract of trophozoites (0, vehicle-DMSO 1% and 150, 215, 300 µM IND), previously incubated 72 h at 37 °C. Results were expressed as a percentage relative to the control (untreated cells set at 100%).

### 4.7. Residual Activity of Recombinant Enzymes GlARr and HsAKR1-A1r

The giardial aldose reductase, recombinant GlARr, and human aldo-keto reductase 1-A1 (HsAKR1-A1r) were purified according to [51], in GlARr with some changes [50], respectively. Enzyme inhibition experiments were carried out in GlARr and HsAKR1-A1r at 0.5 mg/mL with different concentrations of IND in both enzymes (0, vehicle-DMSO 1%, and 150, 215, 300, and 450 μM-IND) in 50 μL, incubated for two and 24 h at 37 °C. The residual activities were monitored in GlARr with 20 μg/mL and HsAKR1-A1r at 5 μg/mL, respectively, in a reaction mix of 0.5 mL in sodium phosphate buffer (pH 7.0) 100 mM, containing MGO 1.75 mM as substrate and cofactor NADP(H) 0.2 mM at 25 °C, due to both enzymes having a methylglyoxal reductase (NADPH) (acetol producing) activity. NADP(H) at 340 nm present in the reaction mix was monitored. The normalized residual activities were taken as a control at 100%. The activity was monitored by following the loss of NADP(H) (ε_340_ = 6.22 mM^−1^·cm^−1^) at 340 nm, which was recorded over time at 25 °C, and enzyme activity was calculated as the rate of NADP formation (ΔAbs_340_/min). The decrease in absorbance at 340 nm was recorded over time, and activities were calculated as ΔAbs_340_/min. All enzymatic assays were performed in duplicate at each time point, and results were expressed as a percentage relative to the control condition (absence of IND), set at 100%.

### 4.8. Indirubin Binding to GlTPIr and GlARr Determines Protein Unfolding by Intrinsic and Extrinsic Fluorescence

The stability and/or unfolding of proteins was analysed using intrinsic and extrinsic fluorescence (FI, EF) in proteins exposed to DMSO or ethanol as a vehicle of IND. The enzyme GlTPIr at 0.2 mg/mL and GlARr at 0.1 mg/mL were incubated previously in 0.8 mL reactions in TE or phosphate buffer, respectively, for 2 and 24 h at 37 °C, including incubation control, vehicle-DMSO 1%, 215 and 300 µM IND.

To determine the effect of vehicle ethanol as an alternative vehicle on GlTPIr, the intrinsic fluorescence spectra of GlTPIr at 0.1 mg/mL in a final volume of 1 mL were incubated alone (control), with IND 450 µM or DMSO vehicle in Trietanolamine-buffer [100 mM]. Fluorescence spectra were recorded at the indicated time points after excitation at 295 nm, with tryptophan signals detected from 300 to 500 nm. At (A) 0 min, (B) 30 min, (C) 90 min, and (D) 150 min. obtained at 25 °C in a Perkin-Elmer LS 55 spectrofluorometer.

The extrinsic fluorescence (EF) was determined using hydrophobic patches exposed on the protein surface by 8-Anilinonaphthalene-1-sulfonic acid (ANSA). At the end of the incubation times, the samples were analyzed in a spectrofluorometer with excitation-emission slits (GlTPI, 2.5-2.5; and GlAR 2.5-3.5) at 0.1 mg/mL of enzyme in 600 µL buffer. Each sample at 166 μg/mL was excited at λ 390 nm, and fluorescence spectra were obtained in a λ 400–600 nm emission scan in the absence and presence of ANSA 160 µM. Spectra were analyzed after subtracting the blank from each sample: (blank, +DMSO 1%, +IND 215 µM, +IND 300 µM, blank +ANSA 160 µM, blank +DMSO +ANSA, blank +IND 215 µM +ANSA, and blank +IND 300 µM +ANSA). The spectral results were plotted, and the peak fluorescence intensity signals at λ 485 nm were compared to obtain the change in signal, which was subsequently normalized to the control [48].

### 4.9. Quantification of Methylglyoxal and Advanced Glycation End Products

Quantification of both MGO and AGEs was conducted using 1 × 10^6^ trophozoites, untreated controls, vehicle-DMSO controls (at 0.5, 0.75, and 1%), and IND-treated (150, 215, and 300 μM). Additionally, the synergistic effect of these compounds (DMSO-IND).

Following incubation for 72 h, trophozoites were centrifuged at 3800 rpm for 12 min at 4 °C, and the pellet was resuspended in PBS, repeating this step three times. Afterward, cells were resuspended at a density of 5 × 10^6^/100 μL in PBS and lysed through three freeze–thaw cycles (15 s in liquid nitrogen/45 s at 37 °C). Aliquots were then withdrawn, and 10% perchloric acid was added to each sample, which was then chilled on ice for 10 min before centrifugation at 12,000 rpm at 4 °C for 10 min. The supernatant was taken and stored at −70 °C until use. Intracellular free MGO was determined spectrophotometrically using 2,4-dinitrophenylhydrazine (DNPH), following the method described by Gilbert and Brandt [54], with modifications by [55]. Before MGO estimation, a standard curve was established using MGO stock solutions (0.1 mM) in distilled water, and 20 mM DNPH in HCl-ethanol (12:88). Various concentrations of MGO (ranging from 0 to 10 μM) were incubated with 0.2 mM DNPH at 42 °C for 45 min.

After incubation, samples were allowed to cool for 5 min at room temperature, and the absorbance of MGO-bis-2,4 dinitrophenylhydrazone was measured at 432 nm using a microplate spectrophotometer. Following this, cell supernatants treated with IND or its combination were used to quantify MGO levels using DNPH-HCl-ethanol. Intracellular free MGO concentrations were estimated from the standard curve, utilizing the extinction coefficient ε = 33,600 M^−1^·cm^−1^ for MGO-bis-2,4-dinitrophenyl-hydrazone. All assays were performed in triplicate, and results are presented as MGO (μM)/1 × 10^6^ cells. Conversely, AGEs were determined using an AGEs ELISA kit according to the manufacturer’s protocol. Aliquots from cell lysates were used to determine protein concentration, which was adjusted to 1 mg/mL, then diluted 1:100 and loaded onto ELISA plates for AGEs concentration determination.

Avidin-peroxidase conjugates were added to the wells, and TMB was used as the staining substrate after thorough washing with PBS. A standard curve was generated using AGEs standards provided in the kit, with concentrations ranging from 0 to 200 ng/mL. Absorbance at 450 nm was measured within the initial 10 min. using an Epoch microplate spectrophotometer reader. Results represent the mean of two independent experiments and are expressed as AGEs (μg/mL).

### 4.10. Western Blotting to Methylglyoxal of Native Proteins Exposed to Indirubin

Alterations of native proteins of *Giardia* due to IND-treatment, previously observed by MGO and AGEs determination, were corroborated by WB vs. MGO in the total extracts. Following, 200 μg of protein was loaded onto SDS-PAGE at 12% to show the formation of adducts. Protein samples of *G. lamblia* trophozoites control and previously incubated with vehicle at 0.5, 0.75 and 1% as well as trophozoites exposed at 150, 215 and 300 μM-IND for 72 h at 37 °C were loaded to gels SDS-PAGE test directed to identify the increment observed due to the formation of adducts and modifications in the extracts; were used to evidence and corroborates the increment of MGO protein adducts.

The protein samples from the different treatments were analyzed, and the presence of ARGp adducts was evidenced by Western blotting using anti-MGO. Briefly, the protein was loaded per lane onto SDS-PAGE, and lane 1 included the Western Protein Standard marker. Electrophoresis was carried out for 0.5 h at 90 V and 65 min at 185 V.

Electrophoretic SDS-PAGE gel, as well as PVDF membranes embedded in absolute methanol and equilibrated in transfer buffer Trizma-base 24 mM; glycine 192 mM; SDS 0.1% and methanol 20% at pH 8.3 for 15 min of shaking. The PVDF membrane was transferred for 1 h at 20 V in a horizontal transfer chamber.

After transfer, the membranes were blocked with Trizma-Base 10 mM; NaCl 150 mM; Tween 20 0.1% (TBS-T) containing 8% BSA at pH 7.6, with constant shaking for 1 h at room temperature. TBS-T buffer was used for 2 washes, each lasting for 10 min. The membranes were incubated in TBS-T (0.1%) + BSA 1% overnight at 4 °C and shaken (10 mL) with 1st Ab anti-methylglyoxal (α-MGO) 1:1000. After incubation, membranes were washed three times with TBS-T at room temperature, with each wash lasting 10 min. Subsequently, 2nd Ab, peroxidase-conjugated anti-mouse IgG, was added in TBS-T at 1:2000 + BSA 1% for 1 h at room temperature, with shaking; three washes with TBS-T for 10 min each were then performed. Detection of target proteins was carried out with luminol in a transilluminator according to [56].

### 4.11. Flow Cytometry Assays

To evaluate the induction of apoptosis-like by IND, trophozoites were cultured for 72 h with an initial inoculum of 5 × 10^4^ cells per tube in TYI-S-33 media with increasing concentrations of IND (150, 215, and 300 µM-IND). A vehicle control group receiving vehicle 0, 0.5, 0.75, 1% DMSO was included to assess the potential cytotoxicity of the vehicle alone.

Following treatment, total cell numbers were quantified using a Neubauer hemocytometer to monitor potential loss of cell viability. For apoptotic analysis, cells were collected, washed twice with cold PBS, and resuspended to a final concentration of 1 × 10^6^ cells/mL in binding buffer (10 mM HEPES pH 7.4, 140 mM NaCl, 2.5 mM CaCl_2_). Annexin V-FITC and Propidium Iodide (PI) staining was performed using a commercial apoptosis detection kit (e.g., from Biolegend), according to the manufacturer’s guidelines.

To establish compensation controls for flow cytometric gating and to distinguish between apoptotic and necrotic populations, additional cell samples were included: (1) untreated and unstained cells, (2) cells treated with 20 µM hydrogen peroxide for 16 h to induce early apoptosis-like, and (3) cells treated with 320 µM hydrogen peroxide for 16 h to induce necrosis. The IND-treatments with vehicle: (4) DMSO 0.5%, (5) 0.75%, and (6) 1%. IND: (7) 150, (8) 215, and (9) 300 μM. These controls were used to define fluorescence spillover and to generate the compensation matrix.

Stained cells were analyzed in a FACs Guava flow cytometer (Millipore), and the data were acquired and processed with InCyte™ Software v3.1. Percentages of viable, early apoptotic, late apoptotic, and necrotic cells were determined based on Annexin V and PI fluorescence profiles. The data are presented as means ± standard deviations from two independent experiments.

Finally, cytometric analysis was performed using a sequential gating strategy. Initially, trophozoites were identified and gated based on forward scatter (FSC-H) and side scatter (SSC-H) parameters to exclude debris and non-cellular events.

For fluorescence analysis, quadrant boundaries (Annexin V-FITC vs. propidium iodide) were established using unstained, Annexin V-only, and PI-only controls. Thresholds for positivity were defined empirically to ensure that ≥95% of unstained cells localized within the double-negative quadrant (Q3) while minimizing signal spillover between FITC and PI channels.

Single-stained controls were used to position the vertical (Annexin V) and horizontal (PI) gates, allowing discrimination between Annexin V-positive and PI-positive populations. This gating strategy enabled classification of viable (Ann^−^/PI^−^), Annexin V-positive (Ann^+^/PI^−^), double-positive (Ann^+^/PI^+^), and PI-positive (Ann^−^/PI^+^) cell populations.

### 4.12. Ultrastructural Modifications by Indirubin in Giardia lamblia

To identify ultrastructural modifications, *Giardia* trophozoites from the experimental and control groups were chilled on ice for 30 min and centrifuged to obtain cell pellets containing more than 10 × 10^6^ trophozoites each. The pellets were fixed with glutaraldehyde 2.5% in 0.1 M PBS (pH 7.2) at 4 °C for 24–48 h and post-fixed with osmium tetroxide 2% in the same buffer for 60 min. After dehydration with increasing concentrations of ethanol and propylene oxide, samples were embedded in EPON (epoxy resin) and polymerized at 60 °C for 24 h. To identify ultrastructural modifications, ultra-thin sections (60–90 nm) were cut using an ultramicrotome (Leica EM UC6) and collected on slot grids covered with formvar membrane. Sections were stained with uranyl acetate and lead citrate. The structural changes from 30 fields for each sample were examined and recorded using a JEM-1011 (JEOL, Osaka, Japan) microscope.

### 4.13. Molecular Docking

Comparative molecular docking analyses were performed to evaluate the structural basis of IND binding to giardial enzymes (GlTPI and GlAR) and their human orthologs (HsTPI and HsAR). Crystallographic coordinates were obtained from the Protein Data Bank (PDB) using the following entries: GlTPI (PDB ID: 4BI6 [57]), HsTPI (PDB ID: 2JK2 [58]), GlAR (PDB ID: 3KRB [51]), and HsAR (PDB ID: 2ALR [59]). Protein structures were prepared by removing crystallographic water molecules and bound ligands. The three-dimensional structure of IND was obtained from the DrugBank database (DB12379) in PDB format. The ligand geometry was inspected and optimized before docking, and rotatable bonds were assigned to allow conformational flexibility during simulations.

Molecular docking was conducted using the CB-Dock server [60] under default parameters. This cavity-detection-guided protocol automatically identifies potential ligand-binding pockets and performs docking calculations using AutoDock Vina 1.2.7 as the internal engine. Grid boxes were therefore defined automatically around the predicted cavities, including the dimer interface region for TPI structures and the NADP(H)-binding cleft for AR structures. Multiple docking poses were generated for each system, and binding energies (kcal/mol) were used to rank ligand conformations. Output complexes were downloaded in PDB format for subsequent analysis.

Cavity volumes were calculated using CavityPlus [61] to evaluate structural permissiveness for ligand accommodation. Representative docking poses were selected based on the lowest binding energies and structural consistency across conformers. Structural visualization and interaction analyses were performed using PyMOL Molecular Graphics System v2.5.0 (Schrödinger, LLC).

### 4.14. Statistical Analysis

The statistical analysis of the data was performed using the NCSS, 2022 (v22.09, Statistical, Graphics, and Sample Size Software). A one-way ANOVA was conducted, followed by Tukey’s post hoc test, with statistical significance defined as *p* < 0.05 (* relative to the corresponding control). The analysis was based on three independent biological replicates, with the mean shown and bars indicating the experimental standard deviation.

## 5. Conclusions

IND acts as a multi-target metabolic inhibitor in *Giardia* by selectively disrupting GlTPI and GlAR activities, leading to glycolytic imbalance, impaired carbonyl detoxification, and apoptosis-like trophozoite death. Structural docking and biochemical assays support preferential effects on parasite enzymes over human orthologs. These findings highlight enzyme-directed metabolic interference as a promising strategy for antigiardial drug development.

Further studies are required to evaluate in vivo efficacy, pharmacokinetics, and host safety, as well as to refine structure–activity relationships that may enhance selectivity and therapeutic potential.

## Data Availability

Data are contained within the article.

## Supplementary Materials

The following supporting information can be downloaded at https://www.mdpi.com/article/10.3390/ijms27104167/s1.

## Abbreviations

The following abbreviations are used in this manuscript:

AGEs	Advanced Glycation End Products
AKR	Aldo-Keto Reductase
AR	Aldose reductase
α-GDH	Alpha-Glycerol 3-phosphate dehydrogenase
ANSA	8-Anilinonaphthalene-1-sulfonic acid
DHAP	Dihydroxyacetone phosphate
DMSO	Dimethylsulfoxide
DNPH	2,4-dinitrophenylhydrazine
ETVp7M	Etch Tobacco Virus Protease 7M
GlAR	*Giardia lamblia* Aldose reductase
GlTPI	*Giardia lamblia* Triosephosphate isomerase
G3P	Glyceraldehyde 3-phosphate
HsAKR1-A1	human Aldo-Keto Reductase-1 A1
HsAR	human Aldo-Keto reductase-1 A1
HsTPI	human Triosephosphate isomerase
IND	Indirubin
LL	Linearolactone
MGO	Methylglyoxal
MT	microtubule
NADP(H)	Reduced Nicotinamide Adenine Dinucleotide Phosphate
NADH	reduced Nicotinamide Adenine Dinucleotide
PBS	phosphate-buffered solution
PI	Propidium Iodide
RGCs	retinal ganglion cells
TBS-T	Trizma-Base-NaCl-Tween-20 buffer
TE	Trietanolamine buffer
TEM	Transmission Electron Microscopy
V-FITC	Annexin V-fluorescein isothiocyanate staining
