# Supplementary material for: Selective Anti-Giardial Action of Indirubin: Biochemical and Functional Evidence for Inhibition of Triosephosphate Isomerase and Aldose Reductase in Giardia lamblia
Source: Int J Mol Sci. 2026 May 7;27(10):4167. doi: 10.3390/ijms27104167 (PMC13206313; doi:10.3390/ijms27104167)
Supplement: Supplementary file 1 [file ijms-27-04167-s001.zip › Supplementary_Data_V3_050526.pdf]

Article

Selective anti-giardial action of indirubin: biochemical and functional evidence for inhibition of triosephosphate isomerase and aldose reductase in *Giardia lamblia*.

Luis Antonio Flores-López [1], Gabriela López-Herrera [2], Yoalli Martínez-Pérez [3], Elías Jaime Matadamas-Ortiz [4], Saúl Gómez-Manzo [5], Gloria Hernández-Alcántara [6], Angélica González-Maciel [7], Rafael Reynoso-Robles [7], Beatriz Hernández-Ochoa [8], Laura Chino-Ríos [9,10], Diego González-Gómez [11], Leonardo Valente Arteaga-Padilla [11], Sergio Enríquez-Flores [12,*] and Ignacio De la Mora-de la Mora [12,*]

1 Laboratorio de Biomoléculas y Salud Infantil, SECIHTI—Instituto Nacional de Pediatría, Mexico City 04530, Mexico; luisbiolexp@gmail.com
2 Laboratorio de Inmunodeficiencias Primarias, Instituto Nacional de Pediatría, Secretaría de Salud, Mexico City 04530, Mexico; lohegabyqbp@gmail.com
3 Tecnológico de Monterrey, Escuela de Medicina y Ciencias de la Salud, Mexico City 14380, Mexico; yoalli.martinez@tec.mx
4 Laboratorio de Investigación de Agro-Recursos y Metabolitos Secundarios, Universidad Autónoma Chapingo, Texcoco 56230, Mexico; emata993@hotmail.com
5 Laboratorio de Bioquímica Genética, Instituto Nacional de Pediatría, Secretaría de Salud, Mexico City 04530, Mexico; saulmanzo@ciencias.unam.mx
6 Departamento de Bioquímica, Facultad de Medicina, Universidad Nacional Autónoma de México, Mexico City 04510, Mexico; ghernandez@bq.unam.mx
7 Laboratorio Morfología Celular y Tisular; Instituto Nacional de Pediatría, Secretaría de Salud, Mexico City 04530, Mexico; agonzalezmaciel@yahoo.com (A.G.-M.); reynosoraf@yahoo.com (R.R.-R.)
8 Laboratorio de Investigación en Ciencias Ómicas y Epidemiología Microbiana, Hospital Infantil de México Federico Gómez, Secretaría de Salud, Mexico City 06720, Mexico; beatrizhb_16@comunidad.unam.mx
9 Posgrado en Ciencias Biológicas (Maestría), Universidad Nacional Autónoma de México, Mexico City 04510, Mexico; laura-cr@ciencias.unam.mx
10 Mexico and Laboratorio de Biomoléculas y Salud Infantil, Instituto Nacional de Pediatría, Secretaria de Salud, Mexico City 04530, Mexico
11 Licenciatura en Bioquímica Diagnóstica, Facultad de estudios superiores Cuautitlán, Universidad Nacional Autónoma de México, Mexico City 04510, Mexico; 318094414@cuautitlan.unam.mx (D.G.-G.); 318173580@cuautitlan.unam.mx (L.V.A.-P.)
12 Laboratorio de Biomoléculas y Salud Infantil, Instituto Nacional de Pediatría, Secretaría de Salud, Mexico City 04530, Mexico
* Correspondence: sergioenriquez@ciencias.unam.mx (S.E.-F.); ignaciodelamora@ciencias.unam.mx (I.D.l.M.-d.l.M.); Tel.: +52-5510840900 (ext. 1425 or 1726) (S.E.-F. & I.D.l.M.-d.l.M.).

Supplementary Table S1. Effect of IND on native enzyme activities of *Giardia lamblia* triosephosphate isomerase and aldose reductase

Compound	Structure	Access	Enzyme Activity		
			Concentration	Inhibition (%)	
				GlTPI	GlAR
			(µM)	Residual activity: µmol·min^{-1}·mg^{-1} (%)	
**Indirubin** (IND) Smile: C1=CC=C2C(=C1)C(=C(N2)O)C3=NC4=CC=CC=C4C3=O	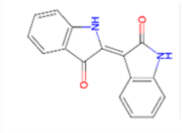	https://www.chemspider.com/Chemical-Structure.4477010.html			
**Dimethylsulfoxide** (DMSO) Smile: CS(=O)C	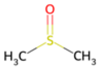	https://www.chemspider.com/Chemical-Structure.659.html	**Control**	**2.975** (64.3 %)	**0.0368** (38.7 %)
			**Vehicle** (DMSO 1 %)	**4.626** (100 %)	**0.0951** (100 %)
			**IND 150**	**1.541** (33.3 %)	**0.0315** (33.1 %)
			**IND 250**	**1.764** (38.1 %)	**0.0334** (35.1 %)
			**IND 300**	**0.383** (8.3 %)	**0.00728** (7.7 %)

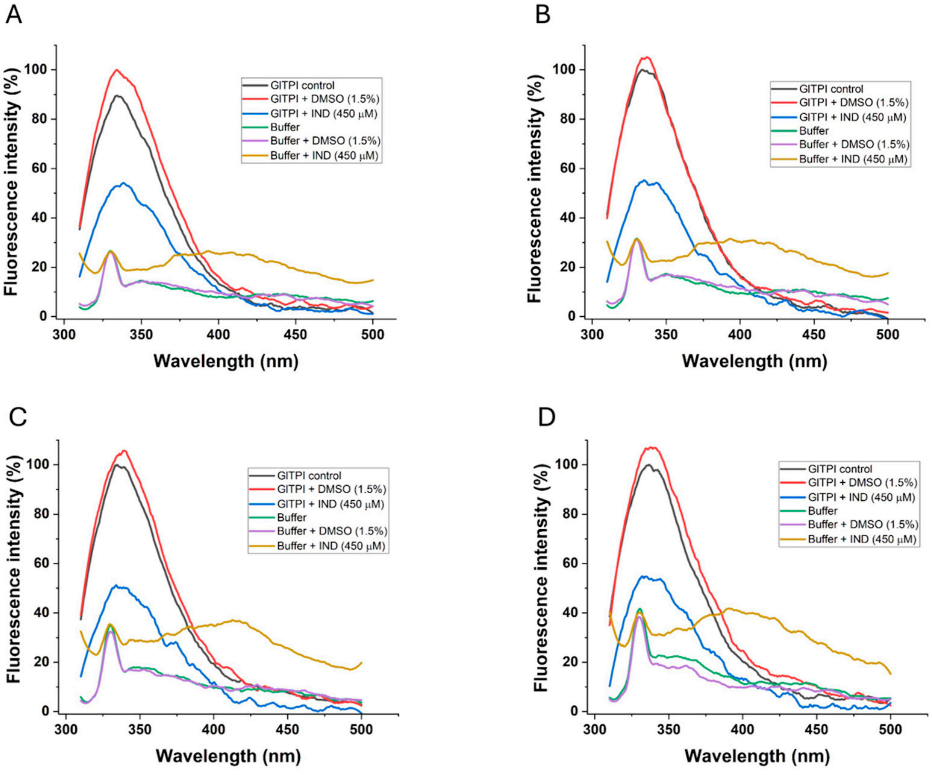

Supplementary Figure S1. Intrinsic tryptophan fluorescence of GlTPIr by IND. Recombinant GlTPI [0.1 mg/mL] was incubated alone (control), with IND [450 µM] or DMSO vehicle in TEA buffer. Fluorescence spectra were recorded at the indicated time points following excitation at 295 nm, with emission detected from 300 to 500 nm. (A) 0 min, (B) 30 min, (C) 90 min, and (D) 150 min.

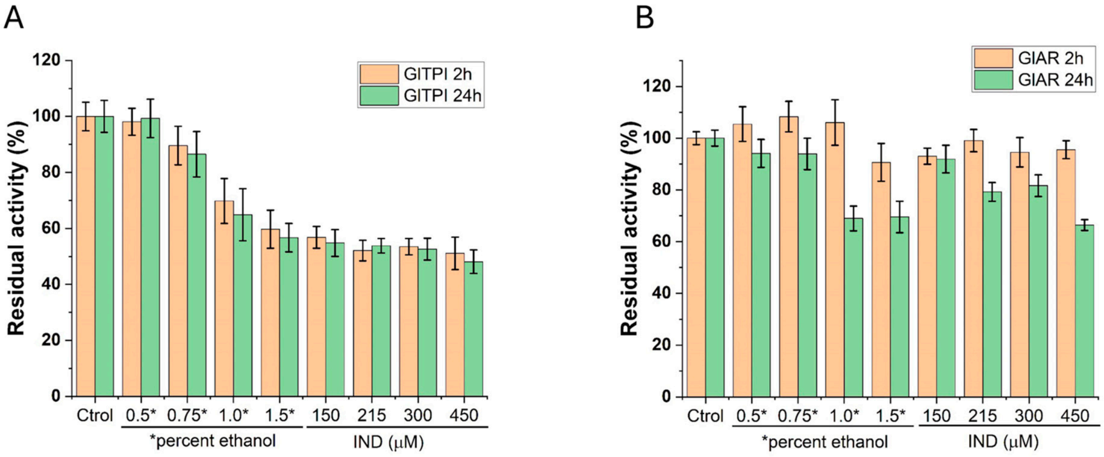

Supplementary Figure S2. Effects of ethanol as an alternative vehicle on GlTPIr and GlARr residual activity in the presence or absence of IND. Recombinant enzymes (0.2 mg/mL) were incubated with increasing concentrations of ethanol alone (0–1.5%) or with IND dissolved in 1.5% ethanol. After the appropriate time (2 h or 24 h), an aliquot was taken and residual enzymatic activity was determined. (A) GlTPIr and (B) GlARr.

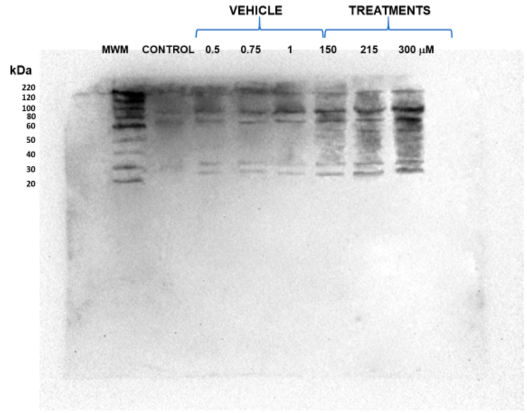

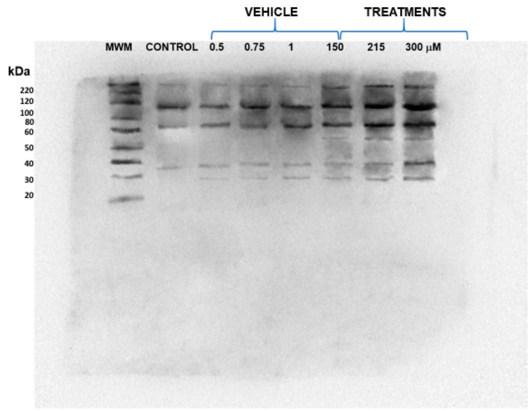

Supplementary Figure S3. Full length of Western blots corresponding to the Figure 7 lower panel *vs.* Methylglyoxal (MGO), shown molecular weight marker (MWM); control and lanes with DMSO vehicle at 0.5, 0.75 and 1% and treatments with IND 150, 215 and 300 μM.

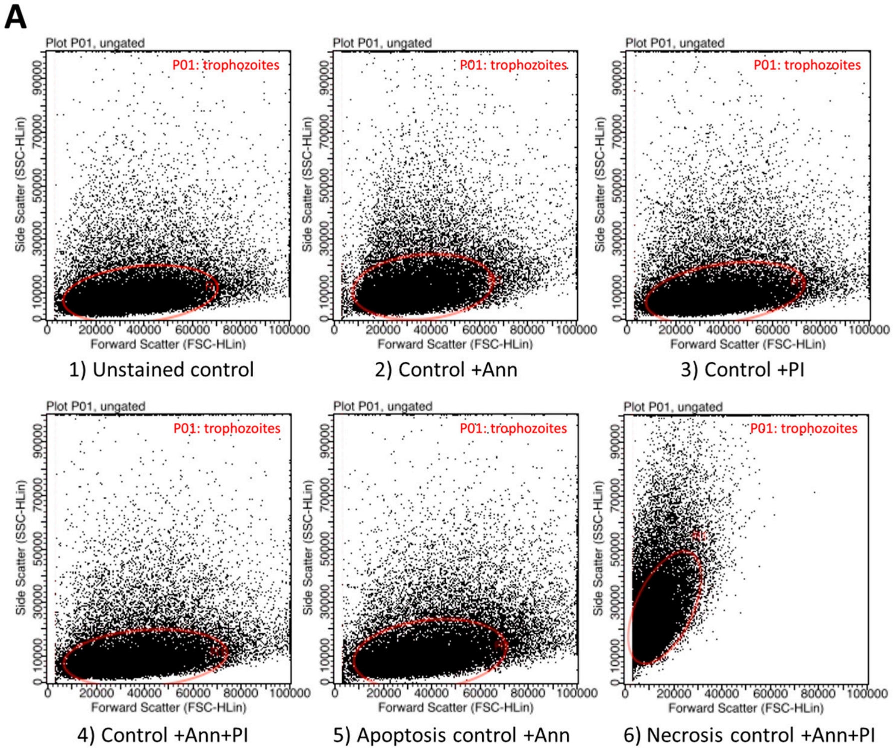

B

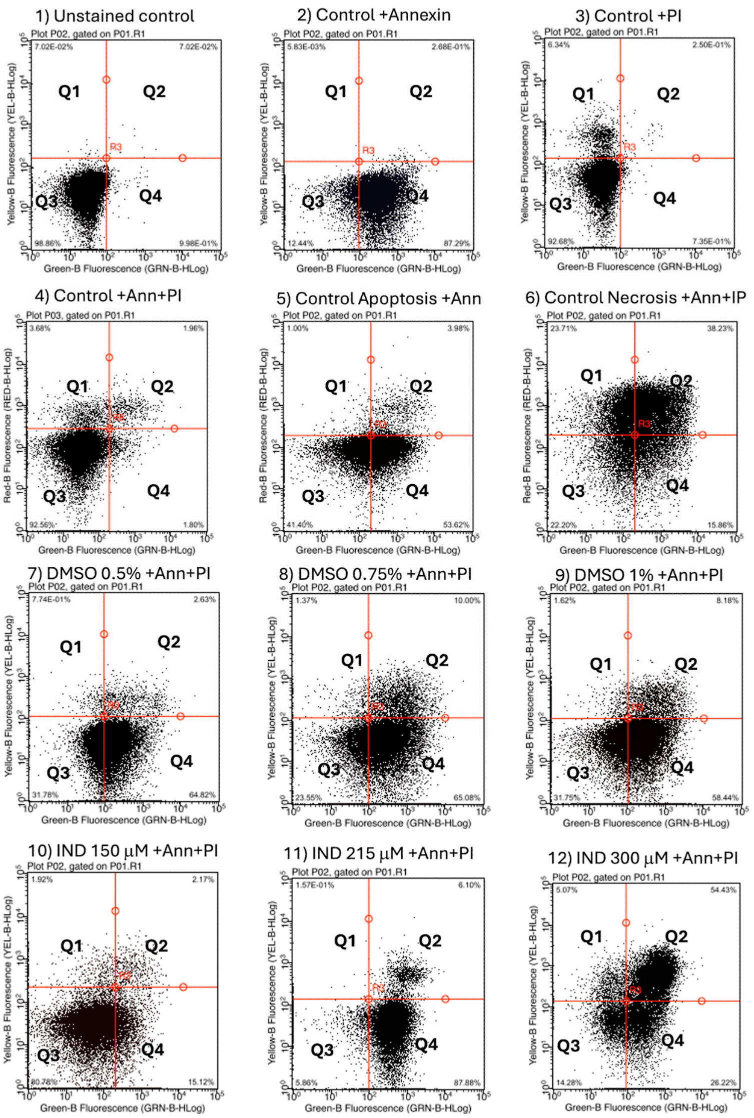

Supplementary Figure S4. Gating strategy validation and representative flow cytometry analysis of IND-treated *G. lamblia* trophozoites. **A)** Gating strategy validation was performed using single-staining and necrosis controls. Dot plots display the fluorescence intensity of Annexin V-FITC (x-axis) versus PI (y-axis), and quadrant boundaries were defined accordingly to discriminate viable, early apoptotic, late apoptotic, and necrotic cell populations. Prior to this analysis, raw cytometry data were examined through forward scatter (FSC) and side scatter (SSC) parameters to assess cell size and granularity, allowing the exclusion of debris and the selection of the main cell population for subsequent fluorescence-based gating. **1) Unstained control**: sets lower-left boundary (autofluorescence) so that >99% of events fall in Q3 (viable, Ann^-/PI^-). **2) Annexin V-FITC alone**: sets vertical boundary between Q3/Q4 and Q1/Q2 at the 99th percentile of the negative population. **3) PI alone**: sets horizontal boundary between Q3/Q1 and Q4/Q2 at the 99th percentile of the negative population. **4) Annexin V + PI (double stain)**: confirms four-quadrant separation. **5) Apoptosis control + Annexin V only**: cells treated with 320 μM H_2O_2 for 16 h, stained only with Annexin V. **6) Necrosis control + Annexin V + PI**: necrosis control with double staining, showing heterogeneous distribution across Q1 and Q2. **B)** representative flow cytometry analysis. Trophozoites were treated with vehicle (0.5%, 0.75%, or 1% DMSO) or IND (150, 215, or 300 μM) for 72 h, then stained with Annexin V-FITC and PI. Quadrants: Q1 (Ann^-/PI^+, necrosis), Q2 (Ann^+/PI^+, late apoptosis/secondary necrosis), Q3 (Ann^-/PI^-, viable), and Q4 (Ann^+/PI^-, early apoptosis). Data are representative of 30,000 events per sample from two independent experiments.

Supplementary Table S2. Flow cytometry analysis of *Giardia lamblia* after IND treatment.

Group	Viability (%)		Early apoptosis (%)		Late apoptosis (%)		Necrosis (%)	
	Mean	(±) S.D.	Mean	(±) S.D.	Mean	(±) S.D.	Mean	(±) S.D.
Control	99.38	0.73539	0.504	0.69862	0.0035	0.00495	0.0035	0.00495
Veh. 0.5%	38.155	6.375	44.49	15.33	9.37	6.74	2.987	2.237
Veh. 0.75%	32.83	9.28	53.835	11.245	10.78	0.784	2.555	1.185
Veh. 1%	36.11	4.36	54.905	3.535	7.2	0.98	1.78	0.16
IND 150 µM	59.865	29.57828	32.3	24.29619	5.705	4.99924	5.705	4.99924
IND 215 µM	12.715	9.69443	47.57	57.00695	25.645	27.6408	25.645	27.6408
IND 300 µM	15.09	1.14551	21.95	6.03869	51.325	4.39113	51.325	4.39113

Values represent the mean and standard deviation (SD) of two independent biological experiments (n = 2).
